# A novel classification method for LUAD that guides personalized immunotherapy on the basis of the cross-talk of coagulation- and macrophage-related genes

**DOI:** 10.3389/fimmu.2025.1518102

**Published:** 2025-02-13

**Authors:** Zhuoqi Li, Ling Chen, Zhigang Wei, Hongtao Liu, Lu Zhang, Fujing Huang, Xiao Wen, Yuan Tian

**Affiliations:** ^1^ Department of Radiotherapy Oncology, Affiliated Hospital of Shandong University of Traditional Chinese Medicine, Jinan, China; ^2^ Department of Oncology, Qingdao Municipal Hospital, Qingdao, China; ^3^ Department of Oncology, The First Affiliated Hospital of Shandong First Medical University & Shandong Provincial Qianfoshan Hospital, Shandong Lung Cancer Institute, Jinan, China; ^4^ Department of Pathology, The First Affiliated Hospital of Shandong First Medical University and Shandong Provincial Qianfoshan Hospital, Shandong Medicine and Health Key Laboratory of Clinical Pathology, Shandong Lung Cancer Institute, Shandong Institute of Nephrology, Jinan, China

**Keywords:** coagulation, macrophage, prognosis, LUAD, classification methods, risk score model, immunotherapy

## Abstract

**Purpose:**

The coagulation process and infiltration of macrophages affect the progression and prognosis of lung adenocarcinoma (LUAD) patients. This study was designed to explore novel classification methods that better guide the precise treatment of LUAD patients on the basis of coagulation and macrophages.

**Methods:**

Weighted gene coexpression network analysis (WGCNA) was applied to identify M2 macrophage-related genes, and TAM marker genes were acquired through the analysis of scRNA-seq data. The MSigDB and KEGG databases were used to obtain coagulation-associated genes. The intersecting genes were defined as coagulation and macrophage-related (COMAR) genes. Unsupervised clustering analysis was used to evaluate distinct COMAR patterns for LUAD patients on the basis of the COMAR genes. The R package “limma” was used to identify differentially expressed genes (DEGs) between COMAR patterns. A prognostic risk score model, which was validated through external data cohorts and clinical samples, was constructed on the basis of the COMAR DEGs.

**Results:**

In total, 33 COMAR genes were obtained, and three COMAR LUAD subtypes were identified on the basis of the 33 COMAR genes. There were 341 DEGs identified between the three COMAR subtypes, and 60 prognostic genes were selected for constructing the COMAR risk score model. Finally, 15 prognosis-associated genes (CORO1A, EPHA4, FOXM1, HLF, IFIH1, KYNU, LY6D, MUC16, PPARG, S100A8, SPINK1, SPINK5, SPP1, VSIG4, and XIST) were included in the model, which was efficient and robust in predicting LUAD patient prognosis and clinical outcomes in patients receiving anti-PD-1/PD-L1 immunotherapy.

**Conclusions:**

LUAD can be classified into three subtypes according to COMAR genes, which may provide guidance for precise treatment.

## Introduction

Lung cancer is a type of cancer with high morbidity, resulting in the most cancer-related deaths worldwide (1). Lung adenocarcinoma (LUAD) is the most common type of lung malignancy and often presents morphologic and genetic diversity, making its diagnosis and treatment difficult (2, 3). Currently, the 5-year survival rate of LUAD patients remains 23%, despite improvements in early diagnosis and current treatment methods (4). In recent years, it has been increasingly recognized that each cancer may have a different response to common treatments, such as chemotherapy and radiation, because of the molecular attributes of an individual patient’s tumor (5). Therefore, further exploration of molecular classification methods for LUAD may contribute to the discovery of more effective therapeutic biomarkers and precise treatments for LUAD.

The tumor microenvironment (TME) plays a crucial role in tumor development and therapeutic response (6, 7). Tumor-associated macrophages (TAMs) are important components of the TME. TAMs mainly originate from two sources: bone marrow (BM)-derived monocytic precursors and tissue-resident macrophages (TRMs) originating from embryonic precursors (8). As the most abundant immune population of the TME, TAMs have heterogeneous properties ranging from antitumorigenic to protumorigenic (9). TAMs can be classified into two types: M1 classically activated macrophages or M2 alternatively activated macrophages (10). The polarization of M1 macrophages is induced by factors such as IFNγ, TNFα, lipopolysaccharide (LPS), GM-CSF, or other pathogen-associated molecular patterns, whereas M2 macrophage polarization is usually stimulated by MCSF, IL4, IL10, IL13, TGFβ, glucocorticoids, or immune complexes (10). M1 macrophages promote an antitumoral response by recruiting Th1 cells through the secretion of the chemokines CXCL9 and CXCL10 and the secretion of proinflammatory cytokines such as TNF-α, IL-1β, IL-6, IL-12 and IL-23 (11, 12). Moreover, M1 macrophages produce nitric oxide (NO) and reactive oxygen intermediates (ROIs), which are toxic to tumor cells (13). M2 macrophages promote tumor progression through the upregulation of immunosuppressive factors such as TGF-β, IL-4, IL-10 and PD-L1 (14, 15) or facilitate tumor angiogenesis via the expression of Tie2, VEGF, PDGF and IGF (15, 16). In addition, M2 macrophages enhance cancer cell drug resistance by regulating the PI3K/Akt, JAK/STAT and mitogen-activated protein kinase (MAPK) pathways through the production and release of mediators (17–19).

M2 macrophages promote lung cancer growth and metastasis through various mechanisms. For example, the IL6-STAT3-C/EBPβ-IL6 positive feedback loop in TAMs promotes the secretion of IL-6, thus facilitating LUAD progression and metastasis by activating the EMT pathway (20). M2 macrophages promote malignancy in lung cancer through EMT by upregulating CRYAB expression and activating the ERK1/2/Fra-1/slug signaling pathway (21). LINC01001 from the exosomes of M2 macrophages can interact with METTL3 and regulate glycolysis in LUAD cells to promote LUAD development (22). TAMs have a strong impact on the clinical outcomes of LUAD patients receiving chemotherapy and PD-1/PD-L1, and studies are exploring TAMs as novel therapeutic targets for LUAD (23–26). Studies have shown that TAMs are closely related to coagulation. First, TAMs can facilitate the coagulation process in cancer patients by producing factor X (FX) and inducing cell-autonomous FXa-PAR2 signaling in cells within the TME (27, 28). In addition, some coagulation-related factors affect tumor progression by regulating the functions of TAMs. For example, plasminogen activator inhibitor-1 (PAI-1) and thrombin can promote the M2 polarization of TAMs in lung cancer and ovarian cancer, respectively (29, 30). Tissue factor (TF) expressed by LUAD cells can recruit TAMs to the TME, promoting the formation of the premetastatic niche (31). Evidence shows that the lungs contribute to platelet biogenesis and are a primary site of terminal platelet production (32); thus, the lungs may play significant roles in coagulation. Lung cancer is an important cause of blood coagulation disorders, as it can result in venous thromboembolism, the second leading cause of cancer patient death (6, 33). Patients with LUAD, which is an independent risk factor for thromboembolism, have a greater risk of venous thromboembolism among lung cancer types (34, 35).

In our previous study, we constructed and validated a coagulation and macrophage-related (COMAR) risk score model for LUAD via bioinformatics methods. This model has effective and robust predictive value for patient prognosis and immunotherapeutic response and provides potential new targets for LUAD treatment (36). In accordance with previous studies, we aimed to further explore molecular classification methods for LUAD on the basis of COMAR genes. These findings may help in the discovery of novel biomarkers for the personalized prediction and treatment of LUAD.

## 2 Materials and methods

### 2.1 Data collection and preprocessing

In this study, we applied identical data collection and preprocessing methods as those used in our previous study (36). We used bulk RNA-seq data and clinical information from the TCGA-LUAD cohort (https://portal.gdc.cancer.gov/projects/TCGA-LUAD) and cohorts from the GEO database (https://www.ncbi.nlm.nih.gov/geo/, GSE30219, GSE37745, GSE41271, GSE42127, GSE50081, GSE68465, and GSE72094). The GSE68465 dataset was used as the training cohort, and the other datasets were used as the validation cohort. The scRNA-seq dataset, which contains single-cell transcriptome data from 15 LUAD patients, was downloaded from the GEO database under accession number GSE131907. Coagulation-related genes were obtained from the coagulation-related pathways in the MSigDB and KEGG databases, with 535 genes identified after the removal of duplicate genes. The numbers of genes in the corresponding pathways and the names of the 535 genes are listed in **Supplementary Table 1**. The preprocessing steps of the scRNA-seq data were also identical to those in our previous study (36). After quality control, batch effect removal, data visualization and cellular group annotation, the differentially expressed genes (DEGs) between each cell type were identified via the “FindAllMarkers” function in the R package “Seurat”, and a volcano plot of the DEGs between different cell types was generated via the R package “scRNAtoolVis”.

### 2.2 WGCNA to construct gene coexpression networks

Weighted gene coexpression network analysis (WGCNA) was used to explore the relationships between the gene coexpression networks and the core genes in the network by identifying coexpressed gene modules. First, the correlation coefficient between every two genes was computed, and the weighted values of the correlation coefficients were used to ensure that the connections between genes in the network obey a scale-free network. A hierarchical clustering tree was subsequently constructed on the basis of the correlation coefficients between these genes. Different branches of the clustering tree represent different gene modules, and different colors represent different modules. Next, the significance of each module was calculated and applied to evaluate the correlation between the M2 macrophage infiltration score and the different modules, and the genes obtained in each module were considered signature module genes.

### 2.3 Estimation of immune cell infiltration in the TME

The CIBERSORT algorithm of the R package “IOBR” was employed to estimate the abundance of 22 types of immune cells in the samples of the GSE68465 cohort. This algorithm can be applied to statistically evaluate the infiltration proportions of cell subgroups in complex tissues according to gene expression profiles, and it is a useful tool for estimating the abundance of specific cell types in mixed tissues.

### 2.4 Unsupervised clustering analysis of coagulation-related genes

The M2 macrophage-related signature genes identified via WGCNA, the TAM-associated DEGs and the coagulation-related genes in the MSigDB and KEGG databases were characterized as key coagulation-related genes. To further reveal the biological functions of the key coagulation-related genes in LUAD, we used the R package “ConsensusClusterPlus” to classify the LUAD patients into different coagulation-related subgroups. The Kaplan−Meier method was used to detect survival status and compare differences in patient survival between these subgroups.

### 2.5 Identification of the DEGs between different coagulation patterns

The R package “limma” was used to identify genes that were differentially expressed between different coagulation patterns. The genes that were differentially expressed between the three coagulation patterns were screened according to the difference multiplicity |log2FC| > 0.585 and the significance threshold FDR (false discovery rate)< 0.05. The DEGs in the three groups were considered coagulation-related genes and were included in the subsequent analyses.

### 2.6 Functional enrichment analysis and construction of the protein interaction network

GO analysis is the main bioinformatic tool for the annotation of genes and their functions and includes three main categories: CC, MF and BP. KEGG is a collection of multiple databases that include information about genomes, biological pathways, diseases and chemicals. GO functional enrichment analysis and KEGG pathway analysis were performed on the DEGs associated with the three coagulation patterns via the “clusterProfiler” package to predict their potential molecular functions. P< 0.05 was considered statistically significant.

### 2.7 Construction of the prognostic model

A 15-gene coagulation-associated prognostic model was constructed on the basis of the DEGs between different coagulation patterns. First, univariate Cox analysis was used to identify the 60 DEGs that were associated with prognosis. Then, LASSO penalized Cox regression analysis was applied to minimize the risk of overfitting, and a 15-gene model was constructed. The LASSO algorithm selected and shrunk the variables via the R package “glmnet”. The patients’ risk scores were calculated on the basis of the expression levels of each prognosis-related gene and their corresponding regression coefficients:


$$\text{Risk score}=\sum_{i=1}^{n}\text{expi}*\;\beta \text{i}$$

In the above formula, “n” represents the number of genes; “expi” represents the expression level of gene “i”; and “βi” represents the coefficient of gene “i”. Patients were divided into high-risk and low-risk groups according to the median risk score, and survival analysis was performed via the R package “survminer” to analyze OS in the high- and low-risk groups. The “survminer” and “timeROC” packages were used to perform time-dependent ROC curve analysis to assess the predictive efficacy of the prognostic models. Finally, risk scores were calculated in the validation cohorts via the same formula.

### 2.8 Pseudotime analysis and cellular communication analysis of the scRNA-seq data

On the basis of the cell annotation results, we selected malignant tumor cells for further analysis. First, the R package “harmony” was used to remove the batch effect among all samples, and the computing method described previously was used to calculate the risk score of each tumor cell. All tumor cells were divided into high- and low-risk groups according to the median risk score. Differential expression analysis of malignant tumor cells was performed for the high- and low-risk groups. We then chose the DEGs in both groups (FDR< 0.05 & |log2FC| > 0.25) for subsequent pseudotime analysis. Next, the “DDRTree” method was used to downscale the cells, and the “reduceDimension” function was used to determine the type of cell differentiation status. Finally, the “plot cell trajectory” function was used to visualize the differentiation trajectory of the cells. The cellular communication between tumor cells in the high- and low-risk score groups and immune cells was analyzed via the R package “cellChat”, and different ligand−receptor pairs were also identified.

### 2.9 Analyses of biological functions

For the bulk RNA-seq data, GO and KEGG enrichment analyses were performed via the GSVA algorithm to calculate the score for each pathway in each sample. The differentially activated pathways in the high- and low-risk score groups were identified via the “limma” package, with the differential threshold set at FDR< 0.05. The classical GO and KEGG analyses were performed via the “clusterProfiler” package for the scRNA-seq data. Differentially activated pathways between the high- and low-risk score groups were analyzed via GSEA enrichment analysis of both bulk RNA-seq and scRNA-seq data.

### 2.10 Collecting the immunotherapeutic cohorts

The GSE126044 dataset, which contains seven LUAD patients who received anti-PD-1 immunotherapy, was downloaded from the GEO database. The GSE135222 dataset containing 27 NSCLC patients receiving anti-PD1/PD-L1 immunotherapy was also downloaded from the GEO database. We calculated the risk scores for each sample in these datasets via the same algorithm as the previous model and performed survival analysis. We also compared the difference in the risk score between patients with cancer progression and those without cancer progression after receiving immunotherapy.

### 2.11 Predicting sensitivity to chemotherapeutic drugs

To assess the differences in sensitivity to chemotherapeutic drugs between the high- and low-risk score groups, the OncoPredict algorithm was used to predict the IC50 of the drugs applied to the samples in the GSE68465 cohort on the basis of the Genomics of Drug Sensitivity in Cancer (GDSC) and the Cancer Therapeutics Response Portal (CTRP) databases. The R package “oncoPredict” was applied to construct the ridge regression model on the basis of the drug data from the GDSC and CTRP databases. Spearman correlation analysis was performed to analyze the correlation between the risk score and drug IC50. In addition, we plotted box plots of the IC50 values of some drugs with differential IC50 values between the high- and low-risk score groups.

### 2.12 Validation of the key COMAR genes at the protein level

The publicly available protein expression data were obtained from the Human Protein Atlas database (https://www.proteinatlas.org/) and the publication of Jun-Yu Xu et al. (37). The immunohistochemical images of key COMAR genes were downloaded from the Human Protein Atlas database, and the protein expression levels of each gene in normal lung tissues and LUAD tissues were observed. The proteomic data generated from mass spectrometry and corresponding clinical information were obtained from the supplemental data of Jun-Yu Xu’s publication (37), and survival analysis was performed on the data.

The key COMAR genes were also validated via immunohistochemical experiments in LUAD clinical samples. The samples were collected from the First Affiliated Hospital of Shandong First Medical University & Shandong Provincial Qianfoshan Hospital from June 2012 to February 2020. Written informed consent was provided by all participants. Tumor tissues were obtained from excised biopsies, fixed in formalin and embedded in paraffin (FFPE) for histological evaluation. After paraffin wax removal and rehydration, the sections were placed in citrate antigen retrieval solution and boiled for 15 minutes for antigen retrieval. Then, an endogenous peroxidase blocker was added to block endogenous peroxidase activity in the sections. After incubation at room temperature for 30 minutes, 50 μL of goat serum working solution was added to each sample, which was subsequently incubated at 37°C for 20 minutes to block nonspecific staining. The sections were subsequently incubated with primary antibodies (rabbit anti-V-set and immunoglobulin domain containing 4 (VSIG4), 1:400, bs-0479R; Bioss Ltd., CHN) for 1 hour at 37°C. After 3 × 5-minute washes with PBS solution, the sections were incubated with biotinylated secondary antibody at room temperature for 30 min, followed by subsequent washes (3 × 5 min in PBS solution). The sections were subsequently dried with absorbent paper and incubated with 50 μL of horseradish-labeled streptavidin for 20 minutes at 37°C. The sections were then rinsed with PBS for 3 × 5 min. After immunostaining, the sections were visualized via the Leica Bond™ System according to the manufacturer’s protocol. The slides were independently examined by two experienced pathologists according to the WHO criteria.

### 2.13 Statistical analysis

All the analyses were performed in R software (version 4.1.2). For significance analysis between various values (such as expression levels, infiltration ratios and various eigenvalues), the Wilcoxon rank-sum test was applied to compare the differences between two groups of samples, and the Kruskal−Wallis test was used to compare the differences between multiple groups of samples. For plot presentation, ns indicates p > 0.05; * indicates p< 0.05; ** indicates p< 0.01; *** indicates p< 0.001; and **** indicates p< 0.0001. Survival curves for the prognostic analysis were generated via the Kaplan−Meier method, and the significance of the differences was determined via the log-rank test.

## 3 Results

### 3.1 Screening the macrophage-related genes involved in this study through WGCNA

This analytical step was aimed at preciously identifying the genes that were significantly related to M2 macrophage infiltration. The CIBERSORT algorithm was used to evaluate the number of M1 and M2 macrophages in the samples from the GSE68465 cohort. The LUAD patients were subsequently divided into M1 and M2 macrophage high and low groups. K−M analysis revealed that there was no significant difference in the survival of LUAD patients between the high-M1 macrophage group and low-M1 macrophage group (**Supplementary Figure 1A**), but patients in the low-M2 macrophage group had longer overall survival (**Supplementary Figure 1B**). These findings suggest that M2 macrophages play an important role in the prognosis of LUAD patients. On the basis of these results, WGCNA was performed to identify M2 macrophage-related genes in LUAD. First, the sample clustering results revealed no outliers in these LUAD samples (**Supplementary Figure 1C**). When the power value was seven, the degree of independence was greater than 0.85 for the first time, so seven was selected as the optimal soft threshold power (**Supplementary Figures 1D, E**). There were nine gene modules identified via WGCNA (**Supplementary Figures 1F, G**). Correlation analysis revealed that genes in the brown module (cor = 0.33, p = 0.0001) and blue module (cor = -0.41, p = 0.0000) were most significantly correlated with M2 macrophages. Therefore, the 408 genes in the brown module and the 430 genes in the blue module (**Supplementary Table 2**) were selected for subsequent analyses.

### 3.2 Acquiring TAM marker genes via scRNA-seq data

The single-cell data was obtained from the study of Nayoung Kim et al, and was downloaded from the GEO database with accession number GSE131907 (38). First, the quality control of the scRNA data was performed for the subsequent analyses (**Supplementary Figures 2A–F**). Then, according to the TSNE and cell type annotation, the cells were divided into two groups: 34279 immune cells and 16236 nonimmune cells. The immune cell group consisted of B lymphocytes, mast cells, myeloid cells, T/NK cells and TAMs, whereas the nonimmune cell group included endothelial cells, epithelial cells and fibroblasts (**Supplementary Figures 3A, B**). The specific genes of each cell type were identified and presented in a volcano plot (**Supplementary Figure 3C**). The 1851 TAM-specific genes were considered TAM-associated genes (**Supplementary Table 3**).

### 3.3 Obtaining the COMAR genes in LUAD

To obtain the genes that were related to the joint functions of coagulation and macrophage. Thirty-three COMAR genes were identified at the intersection of 535 coagulation-related genes, 838 M2 macrophage-associated genes and 1851 TAM-related genes. These genes were selected for subsequent analyses (**Supplementary Figure 4**; **Supplementary Table 4**).

### 3.4 Identification of different COMAR patterns in LUAD

**Figure 1:**
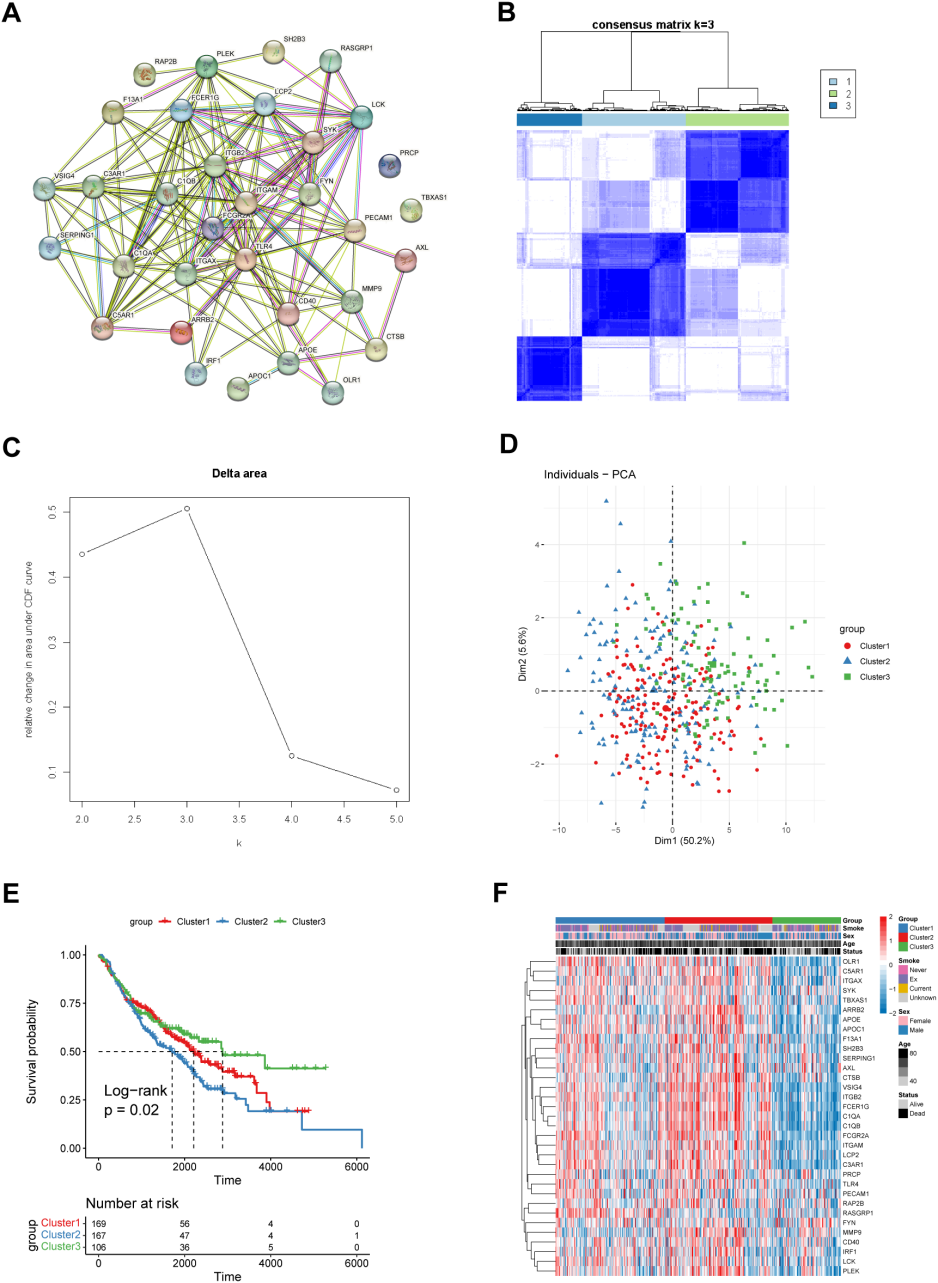
Identification of three different coagulation-associated patterns. **(A)** The PPIN network of the 33 coagulation-related genes constructed via the String database. **(B)** Consensus matrices of the GSE68465 cohort for k = 3. **(C)** Relative change in the area under the CDF curve for k = 2–5. **(D)** Principal component analysis (PCA) of the transcription of the 33 coagulation-related genes in patients with different coagulation patterns. **(E)** OS curves of patients with three different coagulation patterns. Red, cluster 1; blue, cluster 2; green, cluster 3. The abscissa axis shows the survival time, whereas the ordinate axis shows the survival probability. The grouping status of the patients is indicated at the bottom of the chart. P< 0.05 in the log-rank test was considered statistically significant. **(F)** Heatmap showing the expression levels of the 33 genes associated with different coagulation patterns. The three patient groups, smoking status, sex, age and survival status, were used as patient annotations. Red represents high expression of genes, and blue represents low expression.

To further explore the biological and clinical functions of the 33 key coagulation-related genes, we first used the STRING database to construct a protein–protein interaction network (PPIN) to investigate the protein interactions between these genes (**Figure 1A**). The results revealed that many of these genes had strong connections with other genes, such as ITGAM and TLR4. These genes may play important roles in the process of coagulation. We then used the R package “ConsensusClusterPlus” to perform consensus clustering analysis in the GSE68465 cohort. The results revealed that the optimal number of patient subgroups was three (**Figure 1B, C**). The PCA results also revealed three distinct COMAR patterns in these LUAD patients (**Figure 1D**). K−M survival analysis was used to investigate the prognosis of patients in the three COMAR clusters. The three clusters had significantly different prognoses (log-rank test, p = 4.59e-07). Patients in Cluster 3 had the best prognosis, whereas those in Cluster 2 had the worst prognosis (**Figure 1E**). The expression levels of the 33 genes also varied among the three clusters (**Figure 1F**).

### 3.5 Analyzing the DEGs between different COMAR patterns

To explore the underlying mechanisms that caused the differences in biological and clinical functions between the three COMAR patterns, the R package “limma” was used to perform differential expression analysis between the distinct coagulation patterns. A total of 341 genes that were differentially expressed between these clusters were identified with the thresholds set at |log2FC| > 1 and FDR< 0.05. The enrichment analysis for these DEGs was conducted via the R package “clusterProfiler”. The results of the GO analysis revealed that these DEGs were enriched mainly in vesicles and the external side of the plasma membrane and were involved in biological processes such as leukocyte migration, cell adhesion, chemokine receptor binding, MHC protein complex binding, and T-cell activation (**Supplementary Figures 5A–C**; **Supplementary Table 5**). The results of the KEGG enrichment analysis revealed that these DEGs were enriched mainly in signaling pathways such as cell adhesion molecules and phagosomes (**Supplementary Figure 5D**; **Supplementary Table 5**).

### 3.6 Construction and validation of the prognostic model based on the DEGs between different COMAR patterns

**Figure 2:**
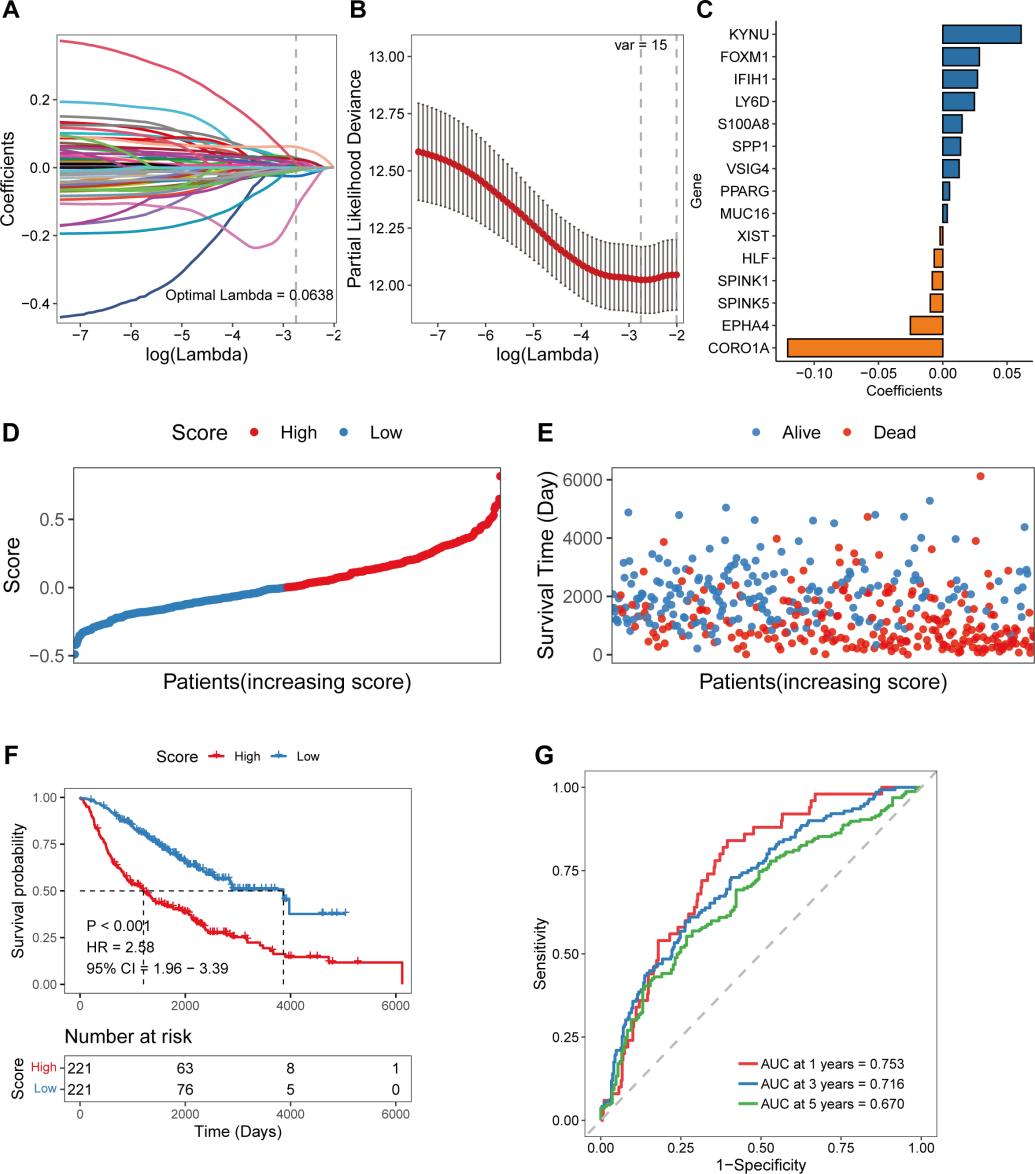
Construction of the coagulation-related 15-gene prognostic model in the training cohort. **(A)** Scatter plot showing the trajectory of each independent variable. The abscissa axis represents the log value of the independent variable lambda. The vertical axis indicates the coefficient of the independent variable. **(B)** The dynamic process diagram of variables screened by LASSO regression analysis and the selection process diagram of the cross-validation parameter lambda. **(C)** Coefficient of each gene included in the prognostic model. **(D)** The risk score distributions of the patients. **(E)** Survival status of the patients. **(F)** The overall survival curve of patients in the high- and low-risk score groups. The abscissa axis shows the survival time, whereas the ordinate axis shows the survival probability. The blue color represents patients with low risk scores, whereas the red color represents patients with high risk scores. The grouping status of the patients is indicated at the bottom of the chart. P< 0.05 in the log-rank test was considered statistically significant. **(G)** ROC curve for predicting the 1-, 3-, and 5-year survival of patients according to the risk score. The abscissa axis represents specificity, and the vertical axis represents sensitivity. Different colors represent different predictive times.

To further investigate the clinical value of the DEGs between different COMAR patterns, a prognostic risk score model was constructed based on these genes. First, univariate Cox regression analysis was performed. There were 60 genes associated with overall survival (OS) (**Supplementary Figure 6A**; **Supplementary Table 6**). KM curves of the top 6 genes with the lowest p values are presented in **Supplementary Figure 6B**. Since an excessive number of genes is not conducive to clinical detection, we then used least absolute shrinkage and selection operator (Lasso) regression analysis to narrow the range of genes involved in the study, and the trajectory of each independent variable was also obtained (**Figure 2A**). As the lambda gradually increased, the number of independent variable coefficients gradually decreased to zero (**Figure 2A**). Tenfold cross-validation was used to build the model, and the confidence intervals under each lambda value are shown in **Figure 2B**. Finally, 15 genes were involved when the model was optimal. Therefore, we selected 15 genes for subsequent analyses and constructed a risk score model according to the coefficients and expression levels of the 15 genes (**Figure 2C**). The calculation formula of the risk score model was as follows:


$$\begin{array}{c} \text{Risk score }=\;\left(-0.006643801\;*\text{ HLF expression level}\right)\\ \;+\;\left(-0.009747989\;*\text{ SPINK}5\text{ expression level}\right)\\ \;+\;\left(0.028388945\;*\text{ FOXM}1\text{ expression level}\right)\\ \;+\;\left(-0.002143226\;*\text{ XIST expression level}\right)\\ \;+\;\left(-0.008215538\;*\text{ SPINK}1\text{ expression level}\right)\\ \;+\;\left(0.012635795\;*\text{ VSIG}4\text{ expression level}\right)\\ \;+\;(0.013814834\;*\text{ SPP}1\text{ expression level}\\ \;+\;\left(0.014965906\;*\text{ S}100\text{A}8\text{ expression level}\right)\\ \;+\;\left(0.026936343\;*\text{ IFIH}1\text{ expression level}\right)\\ \;+\;\left(0.12061942\;*\text{ CORO}1\text{A expression level}\right)\\ \;+\;\left(0.060907371\;*\text{ KYNU expression level}\right)\\ \;+\;\left(0.005199432\;*\text{ PPARG expression level}\right)\\ \;+\;\left(-0.025203004\;*\text{ EPHA}4\text{ expression level}\right)\\ \;+\;\left(0.024547639\;*\text{ LY}6\text{D expression level}\right)\\ \;+\;\left(0.003433631\;*\text{ MUC}16\text{ expression level}\right). \end{array}$$

By using the 15-gene risk score model, the samples in the GSE68465 training cohort were divided into high-risk and low-risk groups according to the median risk score (**Figure 2D**), and a greater proportion of patients in the high-risk group died (**Figure 2E**). Overall survival analysis revealed that the OS of patients in the high-risk group was significantly lower than that of patients in the low-risk score group (log-rank test, p < 0.001) (**Figure 2F**). The ROC curve revealed that the AUCs of the patients at 1, 3, and 5 years were relatively high at 0.753, 0.716, and 0.670, respectively (**Figure 2G**).

**Figure 3:**
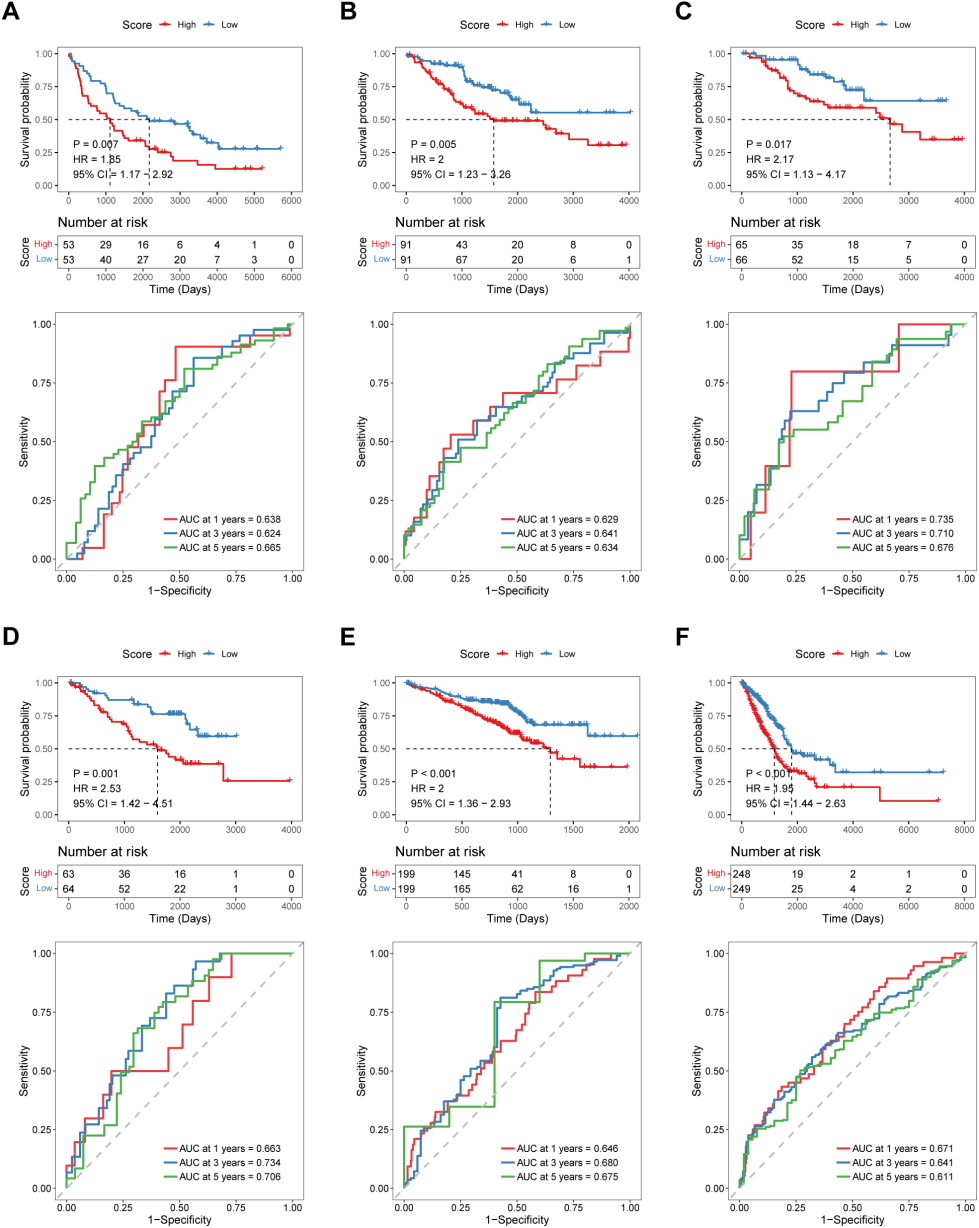
Validation of the predictive efficacy of the 15-gene prognostic model in external independent cohorts: **(A)** GSE37745 cohort, **(B)** GSE41271 cohort, **(C)** GSE42127 cohort, **(D)** GSE50081 cohort, **(E)** GSE72094 cohort, and **(F)** TCGA-LUAD cohort. The upper part of each panel shows the overall survival curve of patients in the high- and low-risk score groups. The abscissa axis shows the survival time, whereas the ordinate axis shows the survival probability. The blue color represents patients with low risk scores, whereas the red color represents patients with high risk scores. The grouping status of the patients is indicated at the bottom of the chart. The lower part of each panel is the ROC curve for predicting the 1-, 3-, and 5-year survival of patients according to the risk score. The abscissa axis represents specificity, and the vertical axis represents sensitivity. Different colors represent different predictive times.

**Figure 4:**
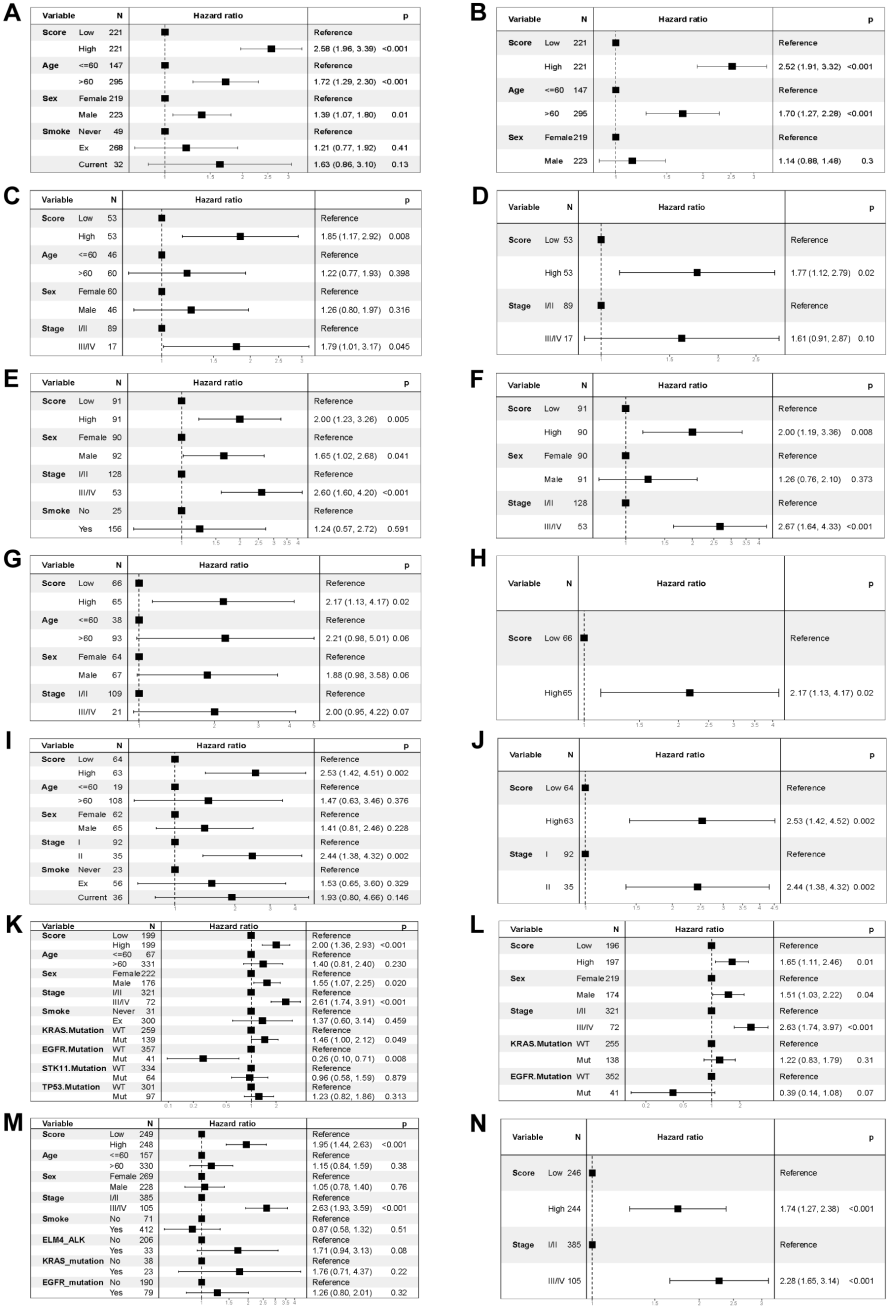
Forest plots of the univariate and multivariate Cox regression analyses for the prognostic model in the training and validation cohorts. **(A)** Univariate Cox regression analysis for the training cohort GSE68465. **(B)** Multivariate Cox regression analysis for the training cohort GSE68465. **(C)** Univariate Cox regression analysis for the validation cohort GSE37745. **(D)** Multivariate Cox regression analysis for the validation cohort GSE37745. **(E)** Univariate Cox regression analysis for the validation cohort GSE41271. **(F)** Multivariate Cox regression analysis for the validation cohort GSE41271. **(G)** Univariate Cox regression analysis for the validation cohort GSE42127. **(H)** Multivariate Cox regression analysis for the validation cohort GSE42127. **(I)** Univariate Cox regression analysis for the validation cohort GSE50081. **(J)** Multivariate Cox regression analysis for the validation cohort GSE50081. **(K)** Univariate Cox regression analysis for the validation cohort GSE72094. **(L)** Multivariate Cox regression analysis for the validation cohort GSE72094. **(M)** Univariate Cox regression analysis for the TCGA-LUAD validation cohort. **(N)** Multivariate Cox regression analysis for the TCGA-LUAD validation cohort. The variables are on the left of each panel. The hazard ratio of each variable and the corresponding forest plot are in the middle of each panel. The p value of the corresponding variable is on the right.

To test the robustness and generalizability of the risk score model constructed on the basis of the 15 COMAR genes in the training cohort, the prognostic efficacy of the risk score model was validated in several external independent datasets via the same algorithm. The results showed that all of the validation cohorts presented results that were consistent with those of the training cohort. The low-risk score group had better overall survival, and the AUCs of the patients all showed high sensitivity and specificity (**Figures 3A−F**). Besides, the risk score model had significantly superior predictive efficacy compared with other clinical factors such as age, sex, tumor stage, and adjuvant chemotherapy at 1-, 3-, and 5-year follow-ups (**Supplementary Figures 7A–I**). To test whether the risk score model is an independent prognostic factor for LUAD patients, we performed univariate and multivariate Cox regression analyses via the “coxph()” function in the R package “survival”. In all the training and validation cohorts, the risk score was an independent prognostic factor among other clinical features, such as age, sex and tumor stage (**Figures 4A−N**). These results demonstrated that the 15-gene prognostic model based on the DEGs between different COMAR patterns possessed strong prognostic efficacy with high robustness and generalizability.

### 3.7 Pseudotime analysis of single-cell RNA-seq data

To further investigate the joint roles of coagulation and macrophage-related genes in single-cell level, a single-cell sequencing dataset (GSE131907) of LUAD patients was used for subsequent analyses. The R package “harmony” was used to eliminate the batch effect of malignant tumor cells between different samples (**Supplementary Figure 8A**). The malignant tumor cells were then divided in a more detailed way such that the tumor cells could be further divided into four different subtypes (**Supplementary Figure 8B**). The percentage stacking plot shows the percentages of the four types of malignant tumor cells in the high- and low-risk score groups. The results revealed that the majority of the cluster 2 subtype was in the low-risk score group, whereas the cluster 0 and cluster 1 subtypes accounted for a greater percentage of the high-risk score group (**Supplementary Figure 8C**). The “FindMarkers” function of the R package “seurat” was used to identify DEGs in the high- and low-risk score groups. A total of 397 DEGs were identified under the conditions of p_val_adj< 0.05 and |avg_log2FC| > 0.25. The UMAP plot shows the calculated risk scores for malignant tumor cells (**Supplementary Figure 8D**). On the basis of the DEGs between the high- and low-risk score groups, we performed simulation analysis for the cellular differentiation trajectory of all malignant tumor cells. In the trajectory plot, the blue color became darker as the cell differentiated earlier, indicating that the tumor cells differentiated from left to right over time (**Supplementary Figure 8E**). We then investigated the differentiation process of tumor cells in the high- and low-risk score groups and found that cells in the low-risk score group differentiated earlier than those in the high-risk score group did (**Supplementary Figure 8F**).

### 3.8 Relationships between the COMAR risk score and the tumor microenvironment

**Figure 5:**
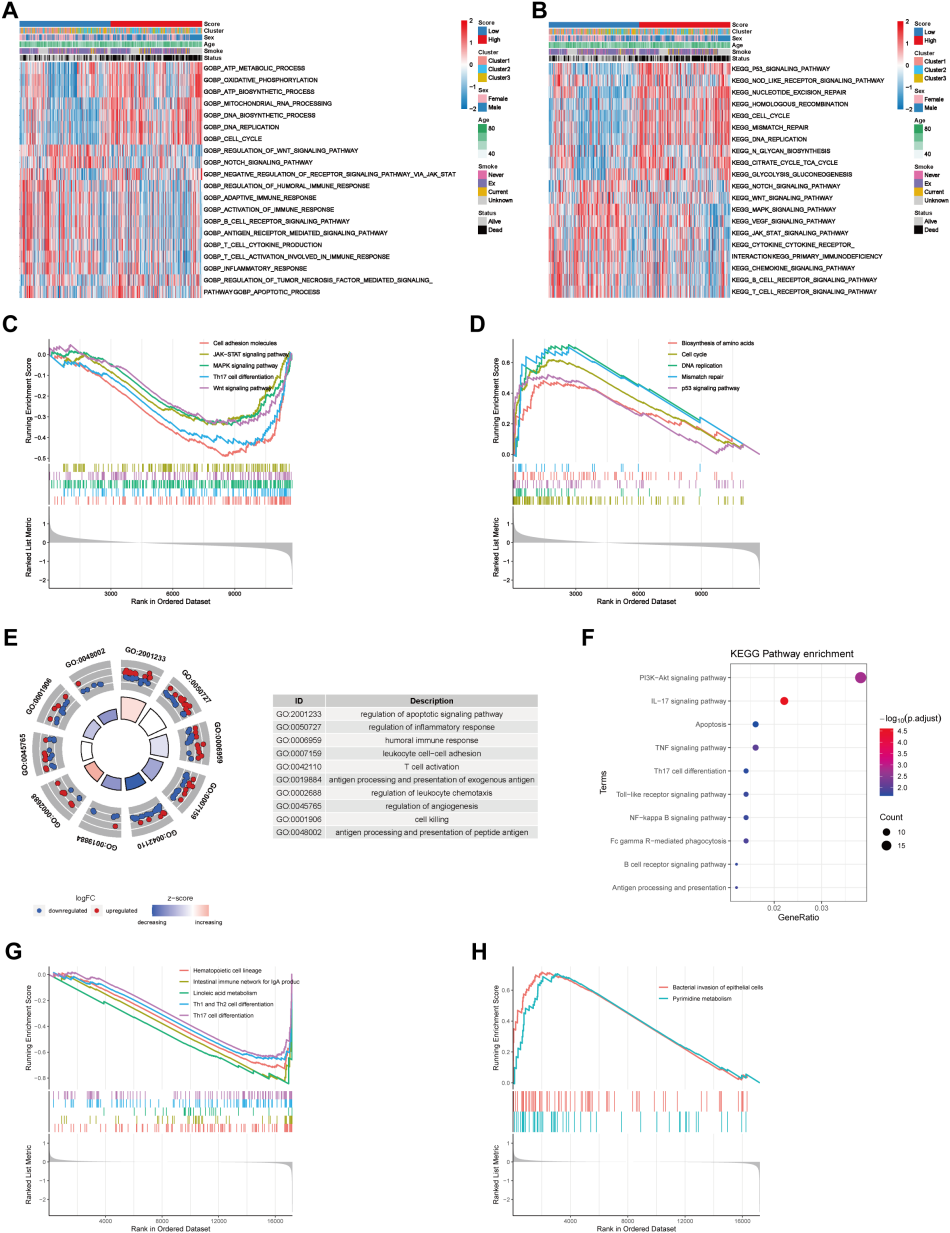
Biological functional analyses of different risk groups on the basis of bulk RNA-seq data and scRNA-seq data. **(A)** GO_BP enrichment analysis of the bulk RNA-seq data via the GSVA algorithm revealed differentially activated biological processes between the low- and high-risk score groups. **(B)** KEGG pathway enrichment analysis of the bulk RNA-seq data via the GSVA algorithm revealed differentially activated biological pathways between the low- and high-risk score groups. The risk score, coagulation cluster, sex, age, smoking status and survival status are used as patient annotations and are at the top of the panels. The biological processes and pathways are listed on the right. Red represents activation, whereas blue represents inhibition. **(C)** Pathways that were activated in the risk score low group in the bulk RNA-seq cohort according to GSEA enrichment analysis. **(D)** Pathways that were activated in the high-risk score group in the bulk RNA-seq cohort according to GSEA enrichment analysis. The abscissa axis represents the ranked gene list according to their expression levels in the two groups. The vertical axis represents the running enrichment score. Curves of different colors represent different pathways. **(E)** GO_BP enrichment analysis of the scRNA-seq cohort via the “clusterProfiler”. **(F)** KEGG pathway enrichment analysis of the scRNA data via the “clusterProfiler”. The left column represents the names of the enriched pathways. The bubbles in the middle column represent the weights of the corresponding pathways, and those in the right column represent the corresponding annotations. **(G)** Pathways that were activated in the low-risk score group in the scRNA-seq cohort according to GSEA enrichment analysis. **(H)** Pathways that were activated in the high-risk score group in the scRNA-seq cohort according to GSEA enrichment analysis.

To research on the relationships between the COMAR risk score model and the TME of LUAD, functional enrichment analyses were conducted on the basis of different risk score groups. We performed GO_BP and KEGG pathway enrichment analyses via the GSVA algorithm in the bulk dataset GSE68465. The results revealed that samples in the high-risk score group were associated with cell proliferation and energy metabolism, whereas samples in the low-risk score group were associated with the activation of immune pathways (**Figure 5A**). For example, the cell cycle, DNA replication and ATP biosynthesis pathways were significantly activated in the high-risk score group, whereas pathways such as B-cell receptor, T-cell activation in the immune response and T-cell cytokine production were significantly activated in the low-risk score group (**Figure 5A**). Moreover, according to the KEGG enrichment analysis, the B-cell receptor signaling pathway, the T-cell receptor signaling pathway, the chemokine signaling pathway, and cytokine and cytokine receptor interactions were activated in the low-risk score group (**Figure 5B**). Similar results were obtained by validating the GO and KEGG enrichment analyses in the scRNA-seq cohort GSE131907. According to the GO enrichment analysis, the DEGs in the high- and low-risk score groups were enriched in pathways such as T-cell activation, the humoral immune response, the inflammatory response, antigen processing and presentation, and apoptosis (**Figure 5E**). Moreover, KEGG enrichment analysis revealed that the differentially expressed genes were enriched mainly in antigen processing and presentation, the B-cell receptor signaling pathway, apoptosis, Th17 cell differentiation, the IL-17 signaling pathway and the TNF signaling pathway (**Figure 5F**). GSEA was also conducted on both bulk RNA-seq and scRNA-seq data. The results of bulk RNA-seq data revealed that pathways such as cell adhesion molecules, Th17 cell differentiation, MAPK, Wnt and JAK-STAT were significantly activated in patients in the low-risk score group (**Figure 5C**). Biosynthesis of amino acids, the cell cycle, DNA replication, mismatch repair and P53 signaling pathways were activated in patients in the high-risk score group (**Figure 5D**). The results of the scRNA-seq data revealed that signaling pathways such as hematopoietic cell lineage, Th1 and Th2 cell differentiation, and Th17 cell differentiation were significantly activated in cells from the low-risk score group (**Figure 5G**). Bacterial invasion of epithelial cells and the pyrimidine metabolism pathway were activated in cells in the high-risk score group (**Figure 5H**). These results indicated that the immune landscape may differ between samples in the high- and low-risk score groups, potentially leading to a diversity of clinical outcomes.

**Figure 6:**
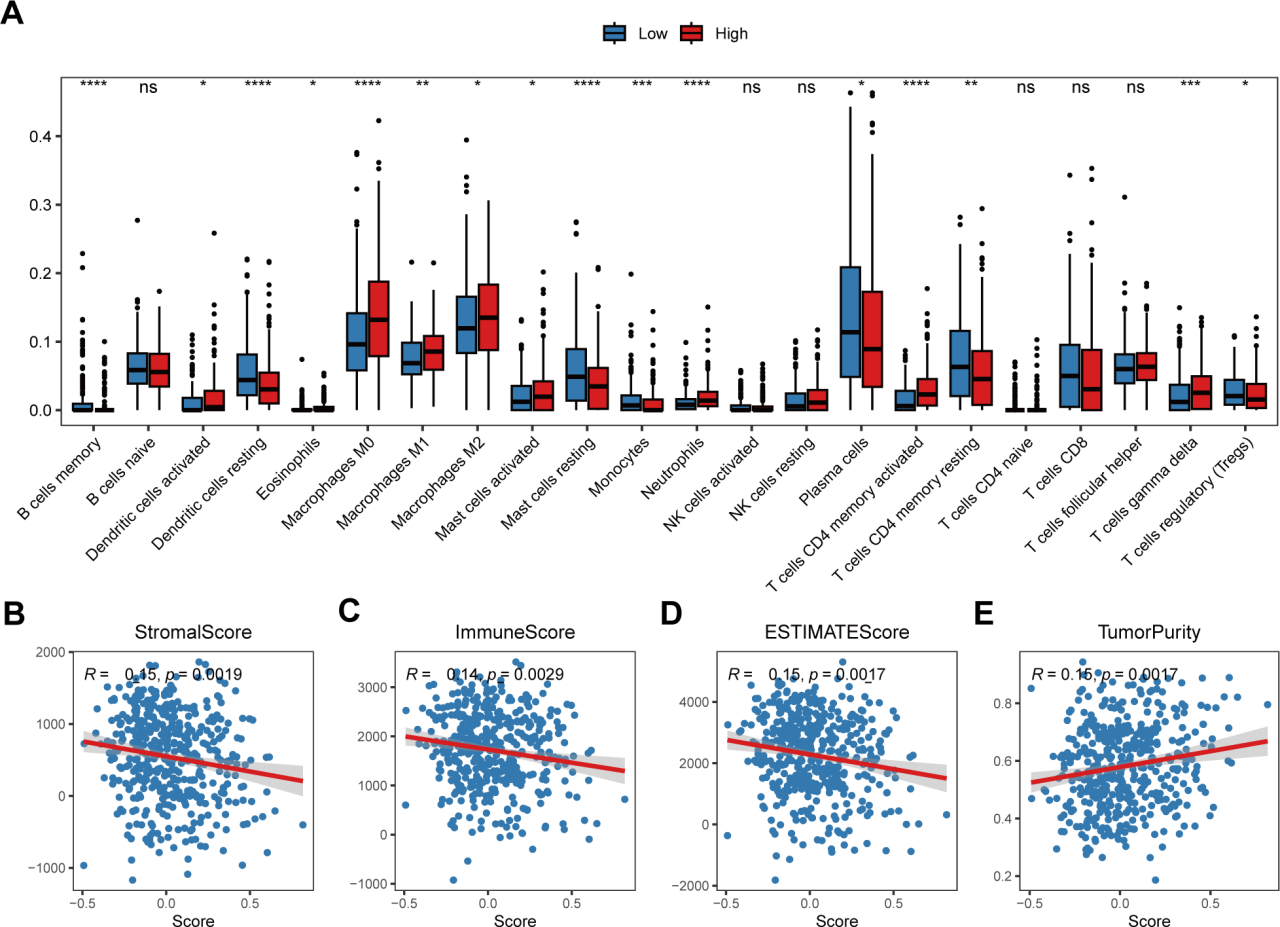
Correlation between the risk score and the tumor immune microenvironment. **(A)** Relative abundances of the 22 types of immune cells in the low-risk score and high-risk score groups. The abscissa axis represents the names of the immune cells. The vertical axis represents the infiltration fraction. **(B)** The correlation between the risk score and the stromal score. **(C)** The correlation between the risk score and immune score. **(D)** The correlation between the risk score and ESTIMATE score. **(E)** The correlation between the risk score and tumor purity. * p< 0.05; ** p< 0.01; *** p< 0.001; **** p< 0.0001; ns, not significant, p> 0.05.

To further explore the correlation between the risk score and tumor immune characteristics, we quantified different immune cell types infiltrating different bulk samples via the CIBERSORT algorithm. The results revealed that immune cell infiltration, such as that of resting dendritic cells, resting mast cells, CD8+ T cells and resting memory CD4+ T cells, was greater in the low-risk score group than in the high-risk score group (**Figure 6A**). However, the infiltration of the three subtypes of macrophages was greater in the high-risk score group (**Figure 6A**). In addition, we analyzed the Spearman correlations between the risk score and the immune score, stromal score, tumor purity, and ESTIMATE score. The results revealed that the risk score was negatively correlated with the immune score, stromal score and ESTIMATE score but positively correlated with tumor purity (**Figures 6B–E**).

### 3.9 Differences in cellular communication between the high- and low-risk score groups based on the prognostic model

Cellular communication is indispensable for the functions of cells in the TME. In this study, cellular communication analysis was performed between immune and tumor cells in the scRNA-seq data via the “CellChat” package. We found that tumor cells in the high-risk group had strong cellular communication with myeloid cells through the GAS signaling pathway, with fibroblasts through the GAS and PERIOSTIN signaling pathways, with endothelial cells through the HSPG and PERIOSTIN signaling pathways, and with T/NK cells through the PAR signaling pathway (**Supplementary Figures 9A–D**).

### 3.10 Value of the COMAR signature in predicting drug sensitivity and clinical outcomes of immunotherapy

**Figure 7:**
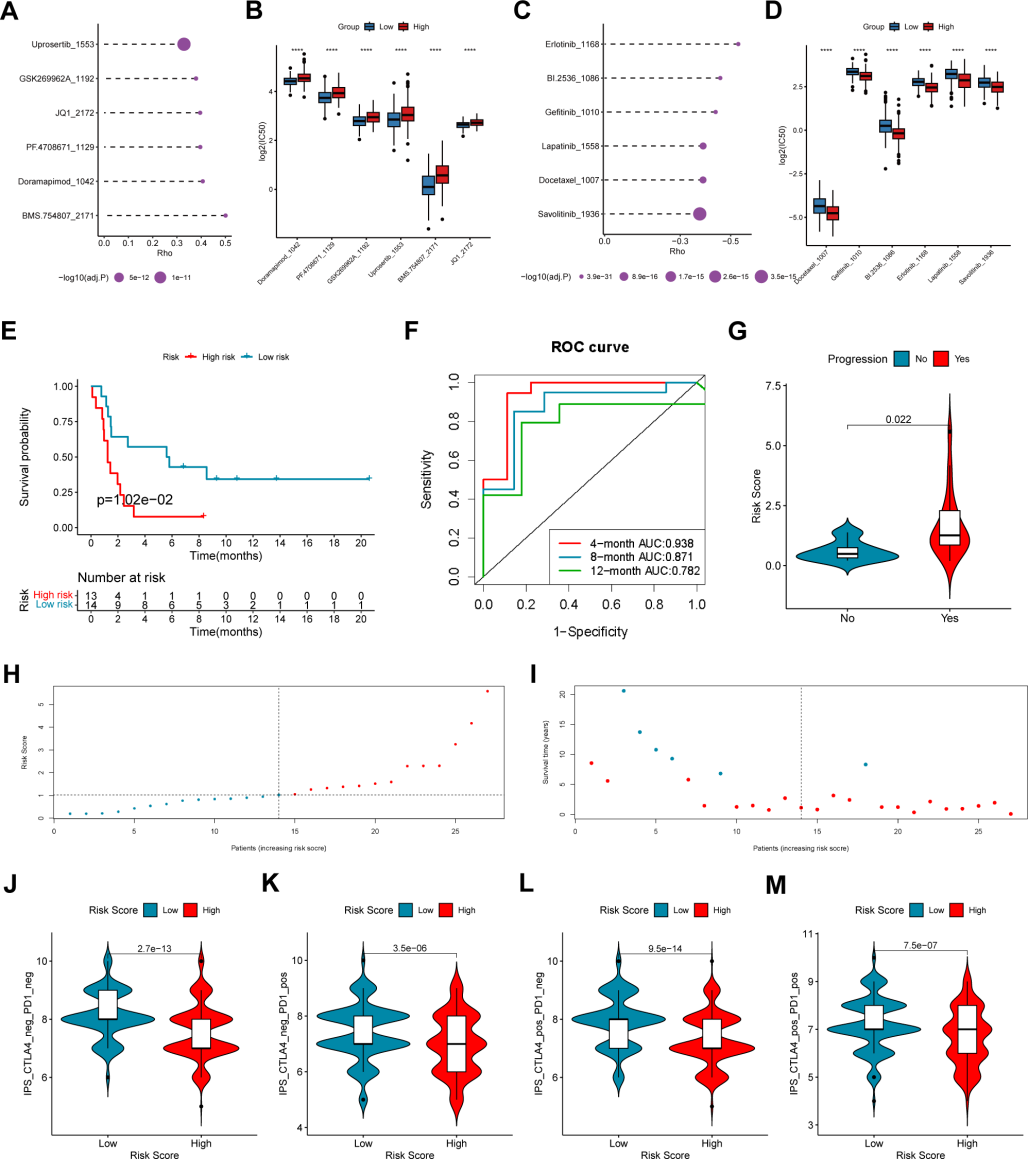
The coagulation-related 15-gene prognostic signature predicts the therapeutic outcomes of patients with LUAD. **(A)** The six drugs that have the highest positive correlation with the risk score. The abscissa axis indicates the correlation coefficient. The vertical axis indicates the names of the six drugs. **(B)** The logIC50 values of the top six positively correlated drugs in the low-risk score and high-risk score groups. The abscissa axis indicates the names of the six drugs. The vertical axis indicates the logIC50 value. Different colors represent different risk score groups. **(C)** The six drugs that have the highest negative correlation with the risk score. The abscissa axis indicates the correlation coefficient. The vertical axis indicates the names of the six drugs. **(D)** The logIC50 values of the top six negatively correlated drugs in the low-risk score and high-risk score groups. The abscissa axis indicates the names of the six drugs. The vertical axis indicates the logIC50 value. Different colors represent different risk score groups. **(E)** The overall survival curve of patients in the high- and low-risk score groups in the anti-PD-1/PD-L1 cohort GSE135222. The abscissa axis shows the survival time, whereas the ordinate axis shows the survival probability. Blue represents patients with low risk scores, whereas red represents patients with high risk scores. The grouping status of the patients is indicated at the bottom of the chart. P< 0.05 in the log-rank test was considered statistically significant. **(F)** ROC curve for predicting the 4-, 8- and 12-month overall survival of patients with LUAD. The abscissa axis represents specificity, and the vertical axis represents sensitivity. Different colors represent different predictive times. **(G)** Violin plot showing the risk score of patients with progression or no progression after anti-PD-1/PD-L1 blockade immunotherapy. **(H)** The risk score distributions of the patients. **(I)** The survival status of the patients. **(J–M)** Correlation analysis between the immunophenoscore (IPS) of anti-cytotoxic T-lymphocyte antigen-4 (CTLA-4) and anti-PD-1 blockade and the risk score in the TCGA-LUAD dataset: **(J)** IPS, **(K)** IPS-PD1, **(L)** IPS-CTLA4, and **(M)** IPS-PD1 + CTLA4. ****, p < 0.0001.

To study whether the COMAR signature have predictive value in LUAD therapy using chemical drugs, the drug IC50 values of the samples in the GSE68465 cohort were predicted via the R package “oncoPredict” and the expression profiles of the drug information in the GDSC database, and the Spearman correlation between the risk score and the drug log2(IC50) was also calculated. The results showed that drugs such as Uprosertib and Doramapimod were significantly positively correlated with the risk score (**Figures 7A, B**), while drugs like Erlotinib and Gefitinib were significantly negatively correlated with the risk score (**Figures 7C, D**).

To explore the predictive efficacy of the 15-gene prognostic model based on the DEGs between different COMAR patterns, we chose the NSCLC cohort GSE135222 treated with anti-PD1/PD-L1 immunotherapy for our analyses. First, we analyzed the relations between the expression of the 15 genes and immune checkpoints in LUAD. The results revealed that the expression levels of some genes, including CORO1A, IFIH1, KYNU and VSIG4 were significantly correlated with those of multiple immune checkpoints (**Supplementary Figure 10**). Subsequently, accordingto the risk score algorithm, the patients were equally divided into risk score high and low groups (**Figure 7H**), and most deaths were in the risk score high group (**Figure 7I**). Survival analysis revealed that patients in the low-risk score group had a superior overall survival status than patients in the high-risk score group did (log-rank test, p = 1.02e-01) (**Figure 7E**). The AUCs of patients at 4 months, 8 months and 12 months were relatively high at 0.938, 0.871 and 0.782, respectively (**Figure 7F**). Patients who experienced progression after immunotherapy also tended to have higher risk scores (**Figure 7G**). In addition, we analyzed the correlation between the immunophenoscore (IPS) and the risk score in the TCGA-LUAD cohort. The results revealed that patients in the low-risk score group had a greater IPS with anti-PD1, anti-CTLA4, and anti-PD1+CTLA4 immunotherapy or without immunotherapy (**Figures 7J–M**), which suggested a better immunotherapy response for patients in the low-risk score group. The above results indicate that the 15-gene prognostic model based on the COMAR patterns has potential value in guiding the clinical treatment of LUAD.

### 3.11 Validation of the results in proteinic data

**Figure 8:**
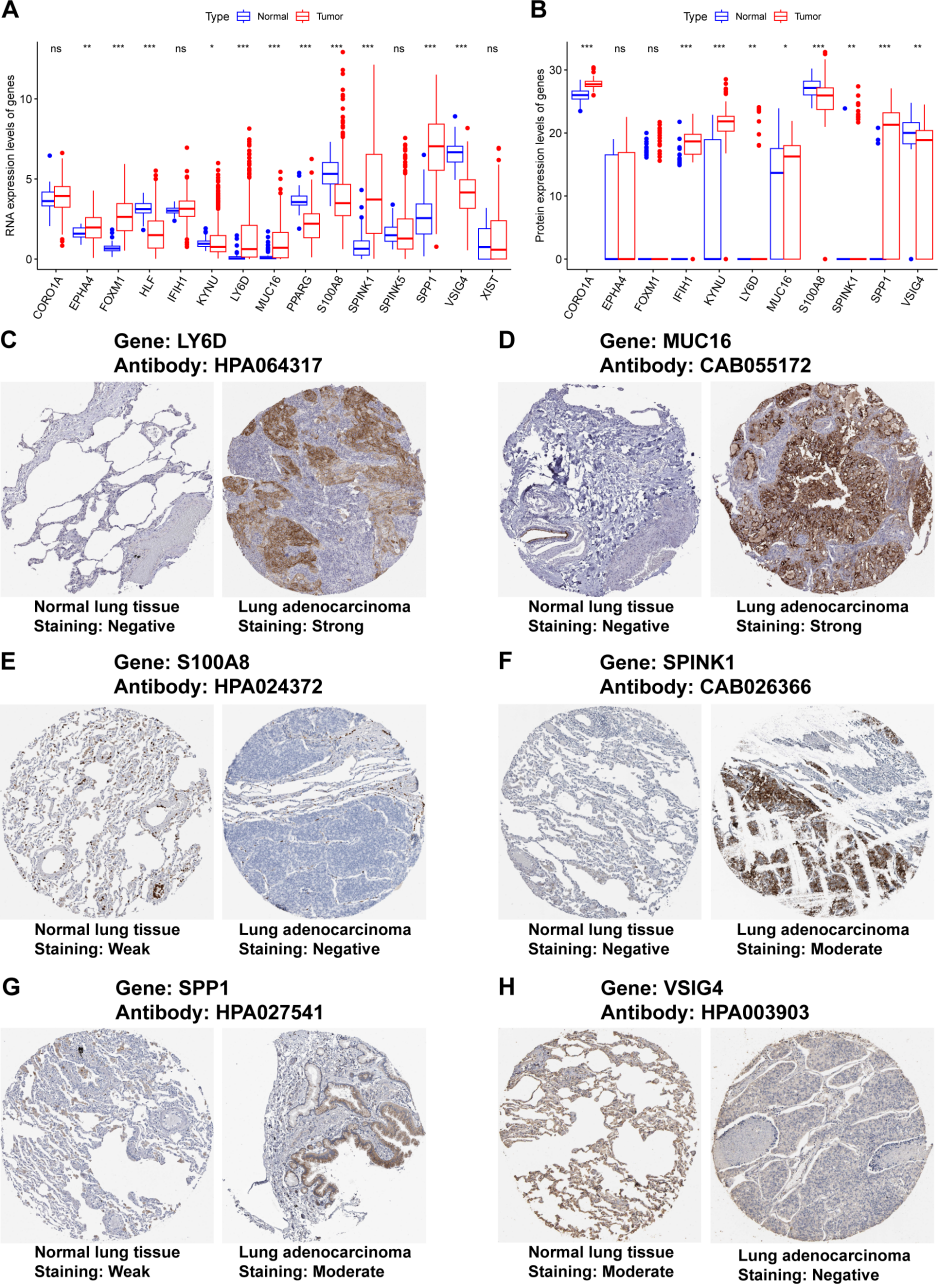
Validation of the expression of the COMAR genes in the prognostic model via proteinic data. **(A)** RNA expression levels of the genes included in the prognostic model in normal lung tissues and LUAD. **(B)** Proteomic expression levels of the genes included in the prognostic model in normal lung tissues and LUAD. The abscissa axis represents the gene names. The vertical axis represents the expression levels. **(C–H)** Immunohistochemical staining images of LY6D **(C)**, MUC16 **(D)**, S100A8 **(E)**, SPINK1 **(F)**, SPP1 **(G)**, and VSIG4 **(H)**. * p< 0.05; ** p< 0.01; *** p< 0.001; ns, not significant, p> 0.05.

**Figure 9:**
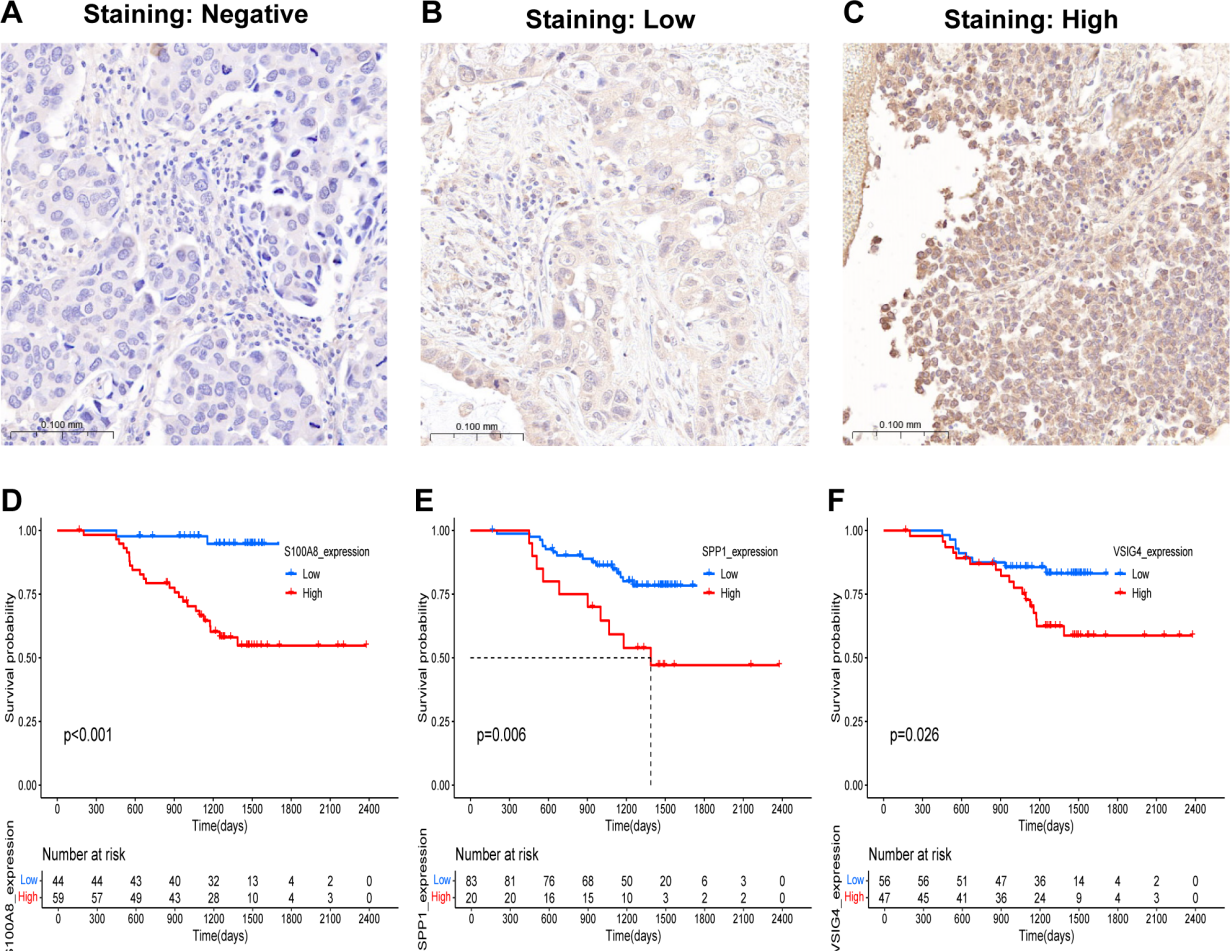
Validation of the predictive value of the COMAR genes in the prognostic model in clinical samples. **(A–C)** Representative images of the immunohistochemical staining intensity of VSIG4: **(A)** negative staining, **(B)** low staining, **(C)** high staining. **(D–F)** Survival curves of LUAD patients in the low- and high-S100A8 **(D)**, SPP1 **(E)**, and VSIG4 **(F)** expression groups. The abscissa axis shows the survival time, whereas the ordinate axis shows the survival probability. Blue represents patients whose genes are expressed at low levels, whereas red represents patients whose genes are highly expressed. The grouping status of the patients is indicated at the bottom of the chart. P< 0.05 in the log-rank test was considered statistically significant.

Our previous results were based on analyses of RNA expression data. To investigate whether the results were reliable at the protein level, the performance of COMAR genes was validated with proteinic data from publicly available databases and our experiments. The results revealed that the expression levels of some COMAR genes involved in the prognostic model were consistent with the RNA expression data (**Figure 8A**), the proteomic expression data (**Figure 8B**) and the immunohistochemical staining intensity (**Figures 8C–H**). For example, LY6D, MUC16, SPINK1 and SPP1 were upregulated (**Figures 8A–D, F, G**), whereas S100A8 and VSIG4 were downregulated in LUAD compared with normal lung tissues (**Figures 8A, B, E, H**). We also performed immunohistochemical staining experiments using clinical LUAD samples, and representative images of negative, low and high staining of the VSIG4 gene are shown in **Figures 9A–C** Among the COMAR genes included in the prognostic model, S100A8, SPP1 and VSIG4 were risk factors for the prognosis of LUAD patients according to the survival analysis of the proteomic data (**Figures 9D–F**).

## 4 Discussion

LUAD is a very complex type of lung malignancy with high morphologic and genetic heterogeneity (2, 3). Individual patients have different therapeutic responses, and the 5-year survival rate of LUAD patients remains low despite improvements in early diagnosis and current treatment methods (4, 5). Precision oncology, which has led to significant advances in the diagnosis and treatment of cancer, is becoming increasingly rapid (5). The development and widespread use of cancer genome analysis has had a great impact on our understanding of the molecular heterogeneity in different cancer patients and has contributed to the development of clinically useful therapeutic agents (5). Therefore, further investigations of the molecular heterogeneity of LUAD may be conducive to precision medicine. The significant roles of the tumor microenvironment (TME) in tumor development and treatment have been revealed by an increasing number of studies (6, 7). As the most abundant immune population of the TME, TAMs strongly affect the progression, metastasis and therapeutic efficacy of LUAD. The functions of TAMs in cancer development are closely related to the coagulation process. TAMs can generate coagulation factors such as factor X (FX), which promotes cell-autonomous FXa-PAR2 signaling in TME cells and results in tumor immune evasion and poor patient prognosis (27, 28). Moreover, some coagulation-related factors can also enhance the tumor-promoting effects of TAMs (29, 30). In our study, a novel molecular classification method for LUAD was developed on the basis of the RNA expression levels of coagulation and macrophage-related (COMAR) genes, and we believe that this classification method may provide guidance for the precision oncology of LUAD.

According to the classification method, LUAD patients can be grouped into three COMAR subtypes (Clusters 1, 2 and 3, **Figures 1B–F**) on the basis of the expression of the 33 COMAR genes. Interestingly, patients in Cluster 3 had significantly better prognoses than those in Clusters 1 and 2 did (**Figure 1E**). Therefore, we intended to further explore the underlying mechanisms that caused the prognostic differences between the clusters. First, we investigated the expression levels of the 33 COMAR genes in the three COMAR clusters (**Supplementary Figure 4**; **Supplementary Table 4**). The results revealed that most of the genes were downregulated in Cluster 3 (**Figure 1F**). Previous studies have suggested that most of these genes, such as OLR1, VSIG4, APOE, APOC1, AXL, CSTB, ITGAM, TLR4, and LCK, are oncogenes, many of which can lead to the progression of LUAD and are associated with poor prognosis (36, 39–45). This finding was consistent with our results (**Figure 1F**).

In the subsequent analyses, DEGs were identified between different COMAR clusters to further explore the biological heterogeneity between COMAR subtypes. Then, functional enrichment analyses were performed for the 341 DEGs (**Supplementary Figures 5A–D**; **Supplementary Table 5**). The results of the GO BP analysis revealed that the DEGs were enriched in biological processes associated with antitumoral immune activation, such as T-cell activation, positive regulation of cytokine production and leukocyte migration (**Supplementary Figure 5A**). Consistently, the GO CC and GO MF analyses suggested that the DEGs were enriched in MHC protein complex formation and binding, immune receptor activity, and cytokine and chemokine activity, which are also related to immune activation (**Supplementary Figures 5B, C**). On the basis of the above results, we speculated that in the COMAR subtype Cluster 3, the biological pathways related to antitumoral immune responses were activated, so patients in Cluster 3 had a better prognosis (**Figure 1E**).

Next, a COMAR subtype-related prognostic signature was constructed on the basis of the 341 DEGs through univariate and LASSO regression analyses. In total, 60 genes were found to be prognostic for LUAD via univariate analysis (**Supplementary Figure 6A**; **Supplementary Table 6**). Finally, 15 genes were included in the COMAR prognostic model via LASSO regression analysis (**Figures 2A–C**). Among the 15 genes, KYUN, FOXM1, IFIH1, LY6D, S100A8, SPP1, VSIG4, PPARG and MUC16 were risk factors for patient prognosis, whereas XIST, HLF, SPINK1, SPINK5, EPHA4 and CORO1A were protective factors (**Figure 2C**). FOXM1 was reported to be associated with poor prognosis in multiple cancers, including LUAD (46). FOXM1 can modulate the expression of PD-L1 in NSCLC cells, which promotes cell proliferation in NSCLC (47). MUC16 facilitates the tumorigenesis and metastasis of NSCLC by regulating TSPYL5 through the JAK2/STAT3/GR axis (48). Moreover, evidence suggests that LY6D, SPP1 and VSIG4 are also associated with NSCLC development and poor prognosis (49–51). There are few reports about the functions of KYUN, IFIH1, S100A8, SPP1 and PPARG in LUAD, which need to be further investigated. Among the protective genes in the prognostic signature, HLF, SPINK5, EPHA4 and CORO1A have been shown to inhibit the proliferation, migration, and invasion of NSCLC cells (52–55). However, XIST and SPINK1 are promoters of NSCLC progression and poor prognosis according to published studies (56–59), which contradicts the results of our study. We will further validate the roles of XIST and SPINK1 in LUAD in future studies.

According to the prognostic model, patients in the training cohort were equally divided into high-risk score and low-risk score groups, and the former had significantly shorter survival times than did the latter (**Figure 2D**). The COMAR model had high prognostic sensitivity and specificity (**Figure 2E**), which were validated in several external datasets (**Figures 3A–F**). The COMAR prognostic signature was also proven to be an independent prognostic factor by univariate and multivariate analyses (**Figures 4A–N**). These findings demonstrated that the COMAR signature had effective and robust predictive efficacy for LUAD patient prognosis.

The COMAR prognostic model could be used to depict the TME in LUAD. The results of GSVA enrichment analysis revealed that some antitumoral immunity-related biological pathways, such as T-cell activation involved in the immune response, the B-cell receptor signaling pathway, the T-cell receptor signaling pathway, and the regulation of the tumor necrosis factor-mediated signaling pathway, were activated in the low-risk score group. Pathways related to cell proliferation, such as the DNA biosynthetic process, DNA replication and the cell cycle, were activated in the high-risk score group (**Figures 5A, B**). The results of the GSEA were consistent with those of the GSVA (**Figures 5C, D**). Similar results were obtained in the enrichment analyses of the scRNA-seq dataset GSE131907. DEGs between LUAD cells in the low-risk score and high-risk score groups were predominantly enriched in pathways associated with immune cell activation and antigen presentation (**Figures 5E, F**). Pathways related to immune cell differentiation were more active in the low-risk group of LUAD cells than in the high-risk group (**Figure 5G**). Immune infiltration analysis revealed that the fraction of infiltrating CD8 T cells, which are tumor suppressive, was greater in the low-risk score group than in the low-risk score group, whereas the proportion of protumoral M2 macrophages was greater in the high-risk score group (**Figure 6A**). The risk score was also negatively correlated with the immune score but positively correlated with tumor purity (**Figures 6C, E**). These results suggest that the activation of the immune response to tumors may be the reason why LUAD patients in the low-risk score group had a superior prognosis.

The discovery and clinical implementation of immune checkpoint inhibitors (ICIs) that target PD-1 and PD-L1 have revolutionized the treatment of cancer. However, the therapeutic effects vary among individuals (60). The COMAR prognostic model showed predictive value for the prognosis of NSCLC patients receiving anti-PD1/PD-L1 immunotherapy (**Figures 7E–I**). Moreover, the COMAR model could also be applied to predict the therapeutic efficacy of small-molecule drugs by analyzing the correlation coefficient between risk scores and drug IC50 values (**Figures 7A–D**). Thus, the COMAR model in our study may provide guidance for personalized ICI immunotherapy and targeted therapy.

The results were validated at the protein level. For validation via RNA expression data from TCGA, proteomic data from the research of Jun-Yu Xu et al. (37), and immunohistochemical staining images from the Human Protein Atlas and our experiments, the expression levels of some genes included in the prognostic model were consistent between normal lung tissues and LUAD (**Figures 8A–H**, **9A–C**). S100A8, SPP1 and VSIG4 were found to be risk factors for LUAD prognosis in the survival analysis via the proteomic data, which was consistent with previous results from the analyses of RNA expression data (**Figures 2C**, **9D–F**). These findings confirmed that the results of our study were reliable.

Certainly, there were limitations in our study. First, our study was based mainly on bioinformatic analyses of public datasets and was only partially verified by experiments on clinical tissues. Biological and molecular experiments *in vitro* and/or *in vivo* are needed to further investigate the functions of the key genes and the activities of the corresponding signaling pathways. Second, owing to the retrospective nature of our study, bias might be inevitable, and prospective experiments are needed for further validation.

## 5 Conclusion

LUAD can be classified into three molecular subtypes on the basis of the cross-talk of coagulation- and macrophage-related (COMAR) genes. A COMAR subtype of LUAD with activation of antitumoral immunity in the TME and a superior prognosis was identified. A 15-gene prognostic signature was constructed on the basis of the DEGs between COMAR subtypes. This signature had high predictive efficacy for prognosis and could depict the TME for LUAD. The novel classification method and key genes identified in our study may contribute to precision oncology for LUAD.

## Data Availability

The original contributions presented in the study are included in the article/**Supplementary Material**. Further inquiries can be directed to the corresponding author.

## Supplementary material

Supplementary Figure 1 Overall survival analysis for the subgroups that contained different abundances of macrophages. **(A)** OS curve for the subgroups with high and low abundances of M1 macrophages. **(B)** OS curve for the subgroups with high and low abundances of M2 macrophages. The abscissa axis shows the survival time, whereas the ordinate axis shows the survival probability. Blue represents low macrophage abundance, whereas red represents high macrophage abundance. The grouping status of the patients is indicated at the bottom of the chart. P< 0.05 in the log-rank test was considered statistically significant. **(C)** Sample clustering in the WGCNA without finding any outliers. **(D, E)** Detecting the optimal soft-thresholding power. When the power value was seven, the degree of independence was > 0.85 for the first time. **(F)** Cluster dendrogram of modular genes associated with M2 macrophage infiltration. Branches of the dendrogram correspond to the different gene modules. Each leaf on the dendrogram represents a gene. Each block marked by a color represents a module that contains a group of highly correlated genes. A total of 10 gene modules were identified. **(G)** Correlations between gene modules and clinical traits. The correlation coefficient and corresponding p value are annotated in the blocks of the module–trait relationship heatmap. Red represents a positive correlation, and blue represents a negative correlation. WGCNA, weighted gene coexpression network analysis.

Supplementary Figure 2 Quality control of the scRNA-seq data. **(A)** The number of genes detected in each cell of each sample. The abscissa axis shows the names of the samples, and the vertical axis shows the number of genes. Each black dot represents a cell. **(B)** The total number of counts in each cell of each sample. The abscissa axis shows the names of the samples, and the vertical axis shows the number of counts. Each black dot represents a cell. **(C)** The percentage of mitochondrial genes in each cell of each sample. The abscissa axis shows the names of the samples, and the vertical axis shows the percentage of mitochondrial genes. Each black dot represents a cell. **(D)** Correlation analysis revealed that the number of genes detected was positively correlated with the depth of sequencing. **(E)** Scatter plot of the top 3000 variable genes. **(F)** The top 20 PCs in the principal component analysis (PCA) for grouping the cells.

Supplementary Figure 3 Acquisition of TAM marker genes. **(A)** The TSNE plot of the samples after removal of the batch effect in the harmony analysis. Different colors represent different samples. The names of the samples are annotated on the right of the plot. **(B)** The TSNE plot of the cells after removal of the batch effect in the harmony analysis. Different colors represent different cell types. The names of the cell types are annotated on the right of the plot. **(C)** Volcano plot showing the genes differentially expressed between different cell types. The red dots indicate upregulated genes, whereas the blue dots indicate downregulated genes.

Supplementary Figure 4 Venn diagram showing the 33 COMAR genes identified from the cross-talk of coagulation-related genes, the M2 macrophage-related genes identified by WGCNA, and the TAM markers. CRGs, coagulation-related genes; WGCNA, weighted gene coexpression network analysis; TAM, tumor-associated macrophage.

Supplementary Figure 5 Enrichment analysis of the DEGs among the three coagulation patterns. **(A)** GO_BP enrichment analysis. **(B)** GO_CC enrichment analysis. **(C)** GO_MF enrichment analysis. **(D)** KEGG pathway enrichment analysis. The left column represents the names of the enriched pathways. The bubbles in the middle column represent the weights of the corresponding pathways, and those in the right column represent the corresponding annotations.

Supplementary Figure 6 Prognosis-related genes among the genes differentially expressed between the three coagulation patterns. **(A)** The forest plot shows the 60 prognosis-related genes. The gene names, hazard ratios and p values of the corresponding genes are on the left of the figure. The corresponding forest plot of these genes is shown on the right of the figure. **(B)** The overall survival curves of the six genes with the lowest p values. The abscissa axis shows the survival time, whereas the ordinate axis shows the survival probability. The blue color represents low expression, whereas the red color represents high expression.

Supplementary Figure 7 The ROC curves of the risk score model and clinical factors at 1, 3, and 5-year follow-up. **(A-C)** ROC analysis of the COMAR risk score, age, sex, and tumor stage on the prognosis at 1-year **(A)**, 3-year **(B)** and 5-year **(C)** follow-up in GSE37745 cohort. **(D-F)** ROC analysis of the COMAR risk score, age, sex, tumor stage and adjuvant chemotherapy on the prognosis at 1-year **(D)**, 3-year **(E)** and 5-year **(F)** follow-up in GSE42127 cohort. **(G-I)** ROC analysis of the COMAR risk score, age, sex, T stage and N stage on the prognosis at 1-year **(G)**, 3-year **(H)** and 5-year **(I)** follow-up in GSE50081 cohort.

Supplementary Figure 8 Pseudotime analysis of the scRNA-seq data. **(A)** The TSNE plot of all the samples after removing the batch effect of malignant tumor cells. Different colors represent different samples. **(B)** The TSNE plot showing different subgroups of malignant tumor cells. Different colors represent different subgroups of tumor cells. **(C)** Percentage chart showing the percentages of different subgroups of tumor cells in the low-risk score and high-risk score groups. **(D)** The TSNE plot showing the calculation results of the risk score for malignant tumor cells. Each dot represents a tumor cell. The red dots represent cells in the high-risk group, whereas the blue dots represent cells in the low-risk group. **(E)** Temporal differences in the differentiation of malignant tumor cells. Different colors represent different pseudotimes. **(F)** Differentiation of cells in the high- and low-risk score groups. Different colors represent different cell groups.

Supplementary Figure 9 Differences in cellular communication between the high- and low-risk score groups. **(A)** Tumor cells in the high-risk score group strongly communicate with myeloid cells and fibroblasts through the GAS pathway. **(B)** Tumor cells in the high-risk score group strongly communicate with endothelial cells through the HSPG pathway. **(C)** Tumor cells in the high-risk score group strongly communicate with T/NK cells through the PAR pathway. **(D)** Tumor cells in the high-risk score group strongly communicate with endothelial cells and fibroblasts through the PERIOSTIN pathway.

Supplementary Figure 10 The correlation analysis between the expression of the 15 genes and immune checkpoints in LUAD.
