# Supplementary figures and images for: A novel classification method for LUAD that guides personalized immunotherapy on the basis of the cross-talk of coagulation- and macrophage-related genes
Source: Front Immunol. 2025 Feb 13;16:1518102. doi: 10.3389/fimmu.2025.1518102 (PMC11866059; doi:10.3389/fimmu.2025.1518102)

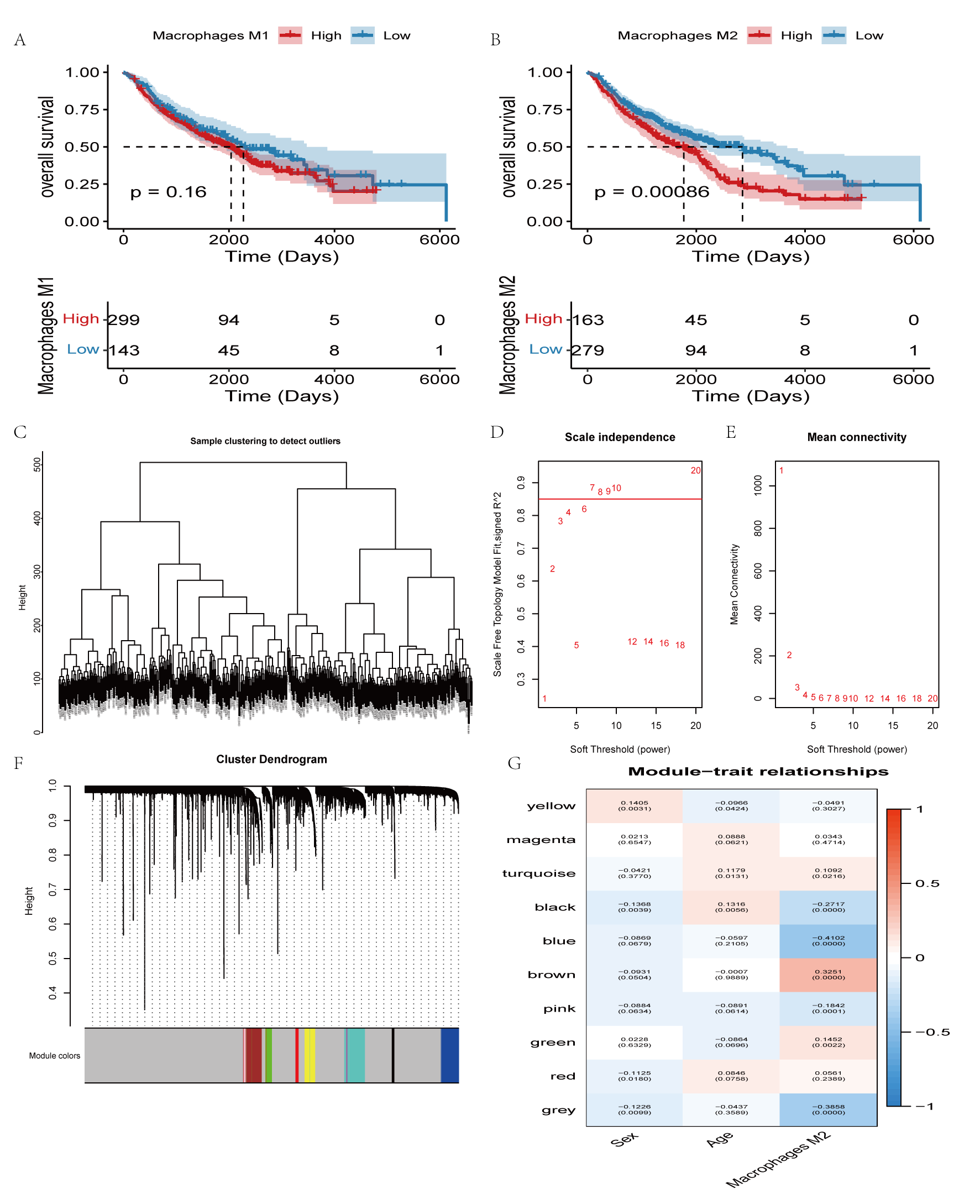

Supplement: Supplementary Figure 1 — Overall survival analysis for the subgroups that contained different abundances of macrophages. (A) OS curve for the subgroups with high and low abundances of M1 macrophages. (B) OS curve for the subgroups with high and low abundances of M2 macrophages. The abscissa axis shows the survival time, whereas the ordinate axis shows the survival probability. Blue represents low macrophage abundance, whereas red represents high macrophage abundance. The grouping status of the patients is indicated at the bottom of the chart. P< 0.05 in the log-rank test was considered statistically significant. (C) Sample clustering in the WGCNA without finding any outliers. (D, E) Detecting the optimal soft-thresholding power. When the power value was seven, the degree of independence was > 0.85 for the first time. (F) Cluster dendrogram of modular genes associated with M2 macrophage infiltration. Branches of the dendrogram correspond to the different gene modules. Each leaf on the dendrogram represents a gene. Each block marked by a color represents a module that contains a group of highly correlated genes. A total of 10 gene modules were identified. (G) Correlations between gene modules and clinical traits. The correlation coefficient and corresponding p value are annotated in the blocks of the module–trait relationship heatmap. Red represents a positive correlation, and blue represents a negative correlation. WGCNA, weighted gene coexpression network analysis. [file Image1.tif]

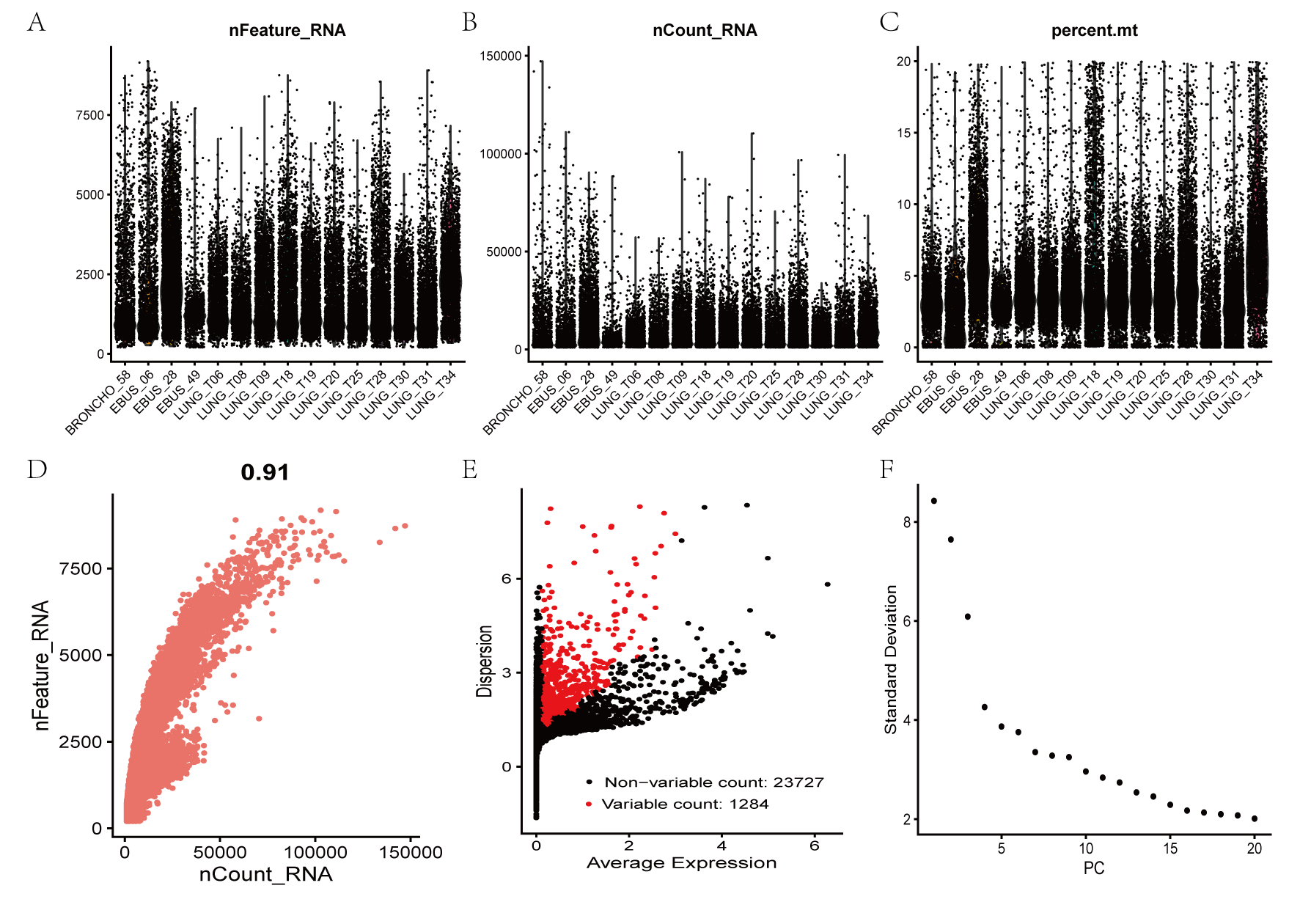

Supplement: Supplementary Figure 2 — Quality control of the scRNA-seq data. (A) The number of genes detected in each cell of each sample. The abscissa axis shows the names of the samples, and the vertical axis shows the number of genes. Each black dot represents a cell. (B) The total number of counts in each cell of each sample. The abscissa axis shows the names of the samples, and the vertical axis shows the number of counts. Each black dot represents a cell. (C) The percentage of mitochondrial genes in each cell of each sample. The abscissa axis shows the names of the samples, and the vertical axis shows the percentage of mitochondrial genes. Each black dot represents a cell. (D) Correlation analysis revealed that the number of genes detected was positively correlated with the depth of sequencing. (E) Scatter plot of the top 3000 variable genes. (F) The top 20 PCs in the principal component analysis (PCA) for grouping the cells. [file Image2.tif]

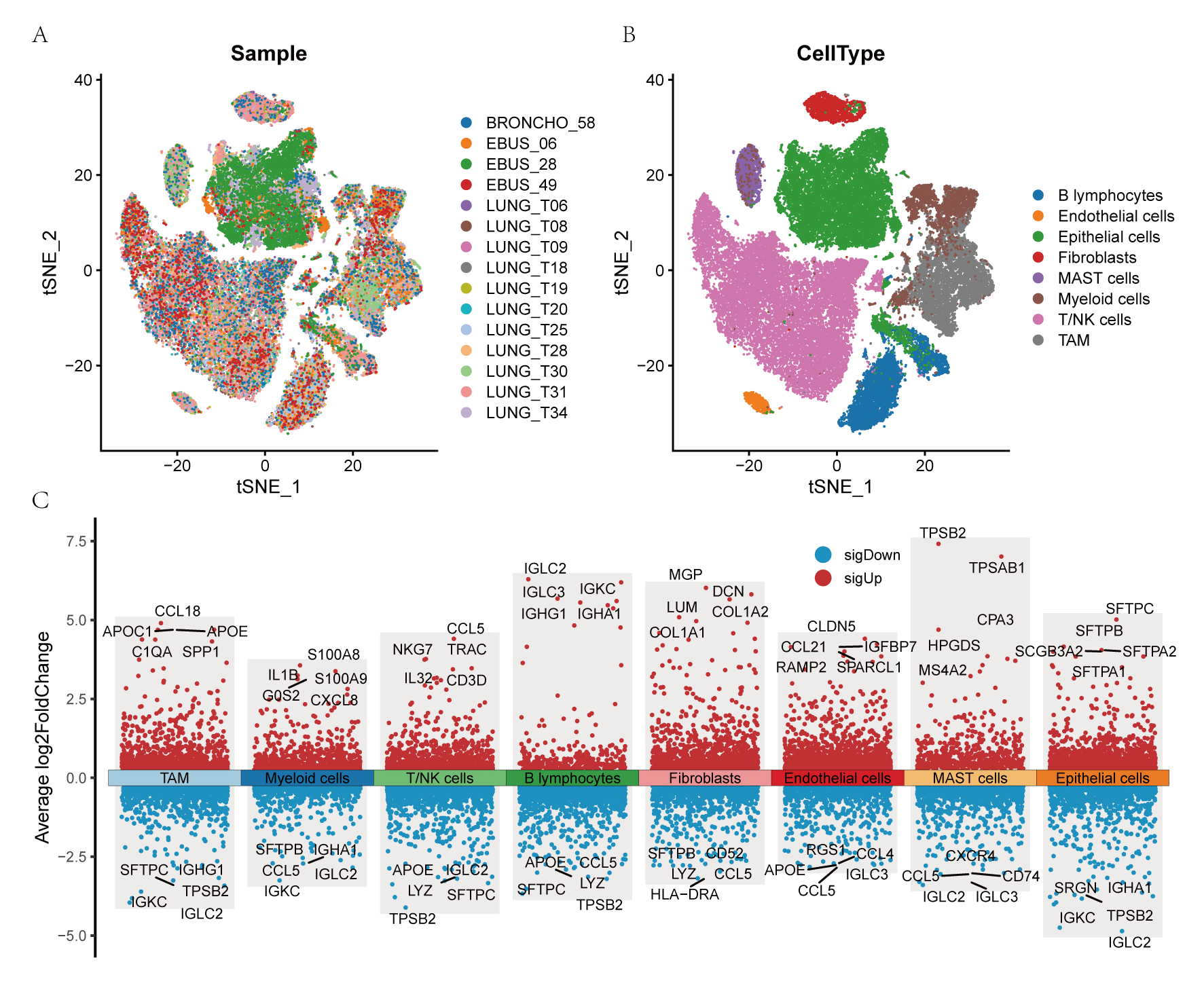

Supplement: Supplementary Figure 3 — Acquisition of TAM marker genes. (A) The TSNE plot of the samples after removal of the batch effect in the harmony analysis. Different colors represent different samples. The names of the samples are annotated on the right of the plot. (B) The TSNE plot of the cells after removal of the batch effect in the harmony analysis. Different colors represent different cell types. The names of the cell types are annotated on the right of the plot. (C) Volcano plot showing the genes differentially expressed between different cell types. The red dots indicate upregulated genes, whereas the blue dots indicate downregulated genes. [file Image3.tif]

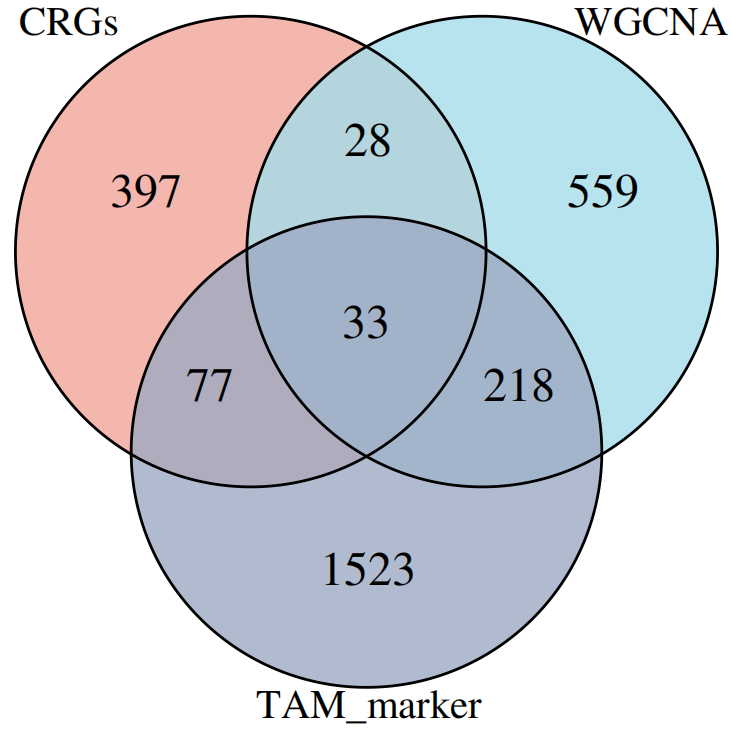

Supplement: Supplementary Figure 4 — Venn diagram showing the 33 COMAR genes identified from the cross-talk of coagulation-related genes, the M2 macrophage-related genes identified by WGCNA, and the TAM markers. CRGs, coagulation-related genes; WGCNA, weighted gene coexpression network analysis; TAM, tumor-associated macrophage. [file Image4.tif]

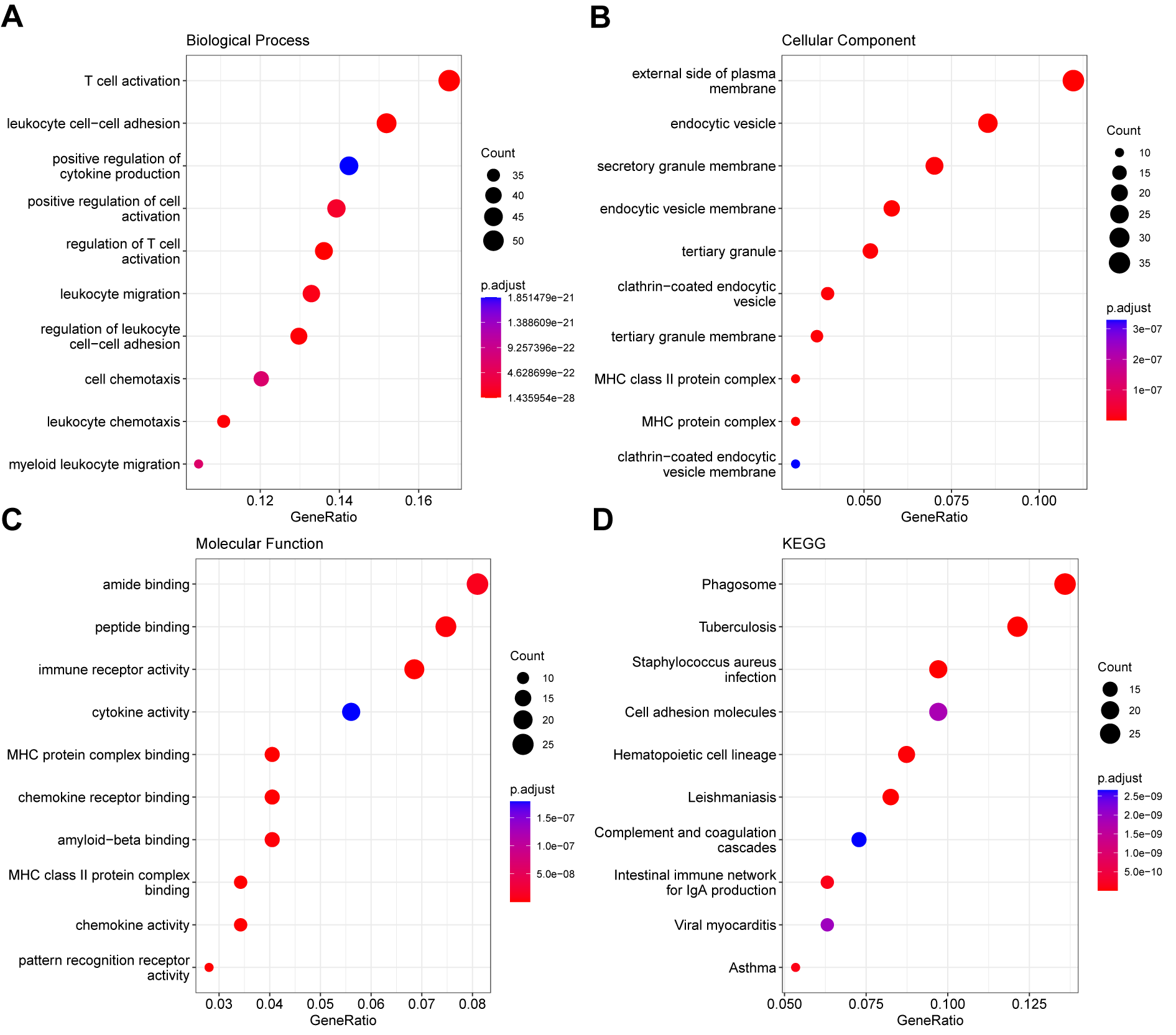

Supplement: Supplementary Figure 5 — Enrichment analysis of the DEGs among the three coagulation patterns. (A) GO_BP enrichment analysis. (B) GO_CC enrichment analysis. (C) GO_MF enrichment analysis. (D) KEGG pathway enrichment analysis. The left column represents the names of the enriched pathways. The bubbles in the middle column represent the weights of the corresponding pathways, and those in the right column represent the corresponding annotations. [file Image5.tif]

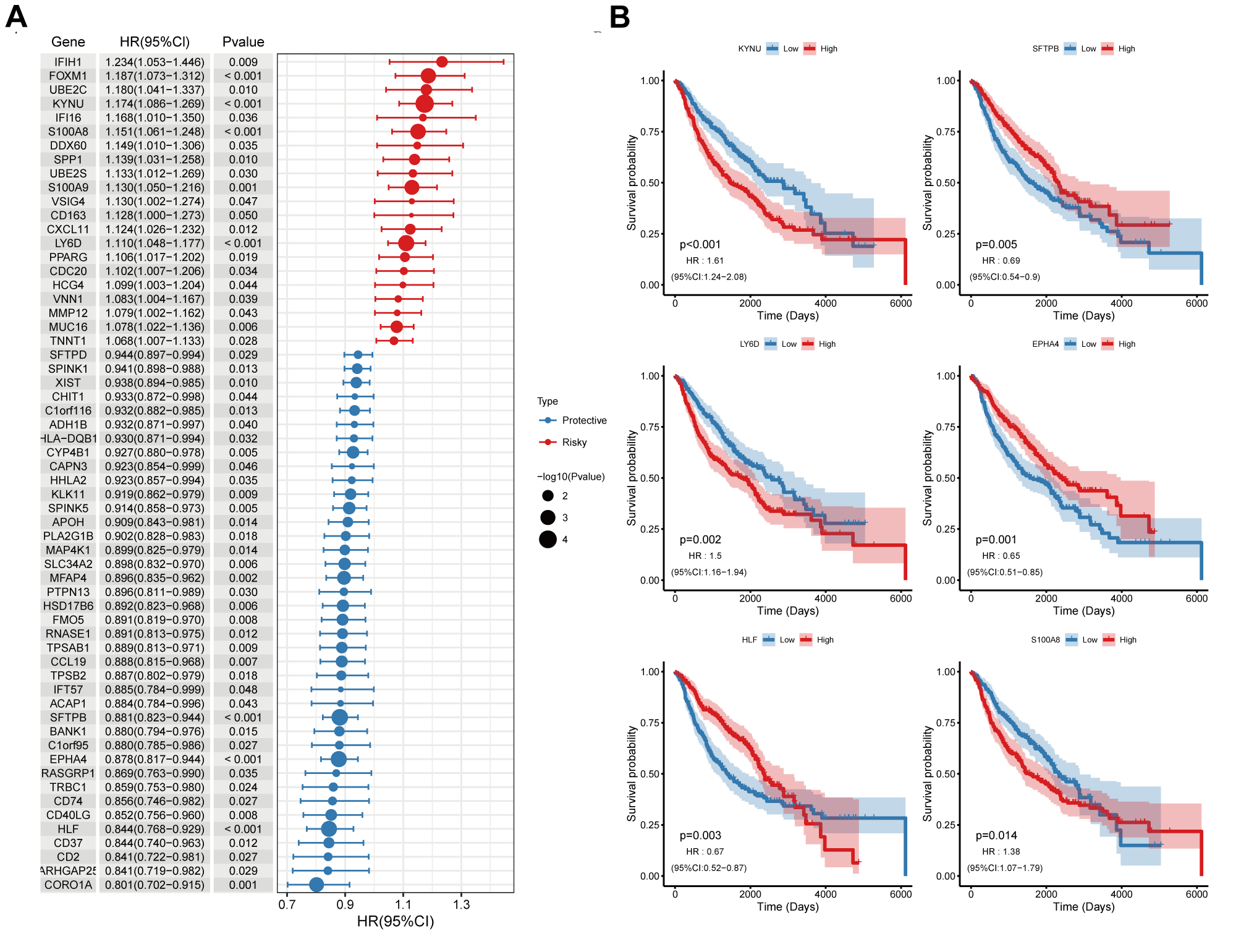

Supplement: Supplementary Figure 6 — Prognosis-related genes among the genes differentially expressed between the three coagulation patterns. (A) The forest plot shows the 60 prognosis-related genes. The gene names, hazard ratios and p values of the corresponding genes are on the left of the figure. The corresponding forest plot of these genes is shown on the right of the figure. (B) The overall survival curves of the six genes with the lowest p values. The abscissa axis shows the survival time, whereas the ordinate axis shows the survival probability. The blue color represents low expression, whereas the red color represents high expression. [file Image6.tif]

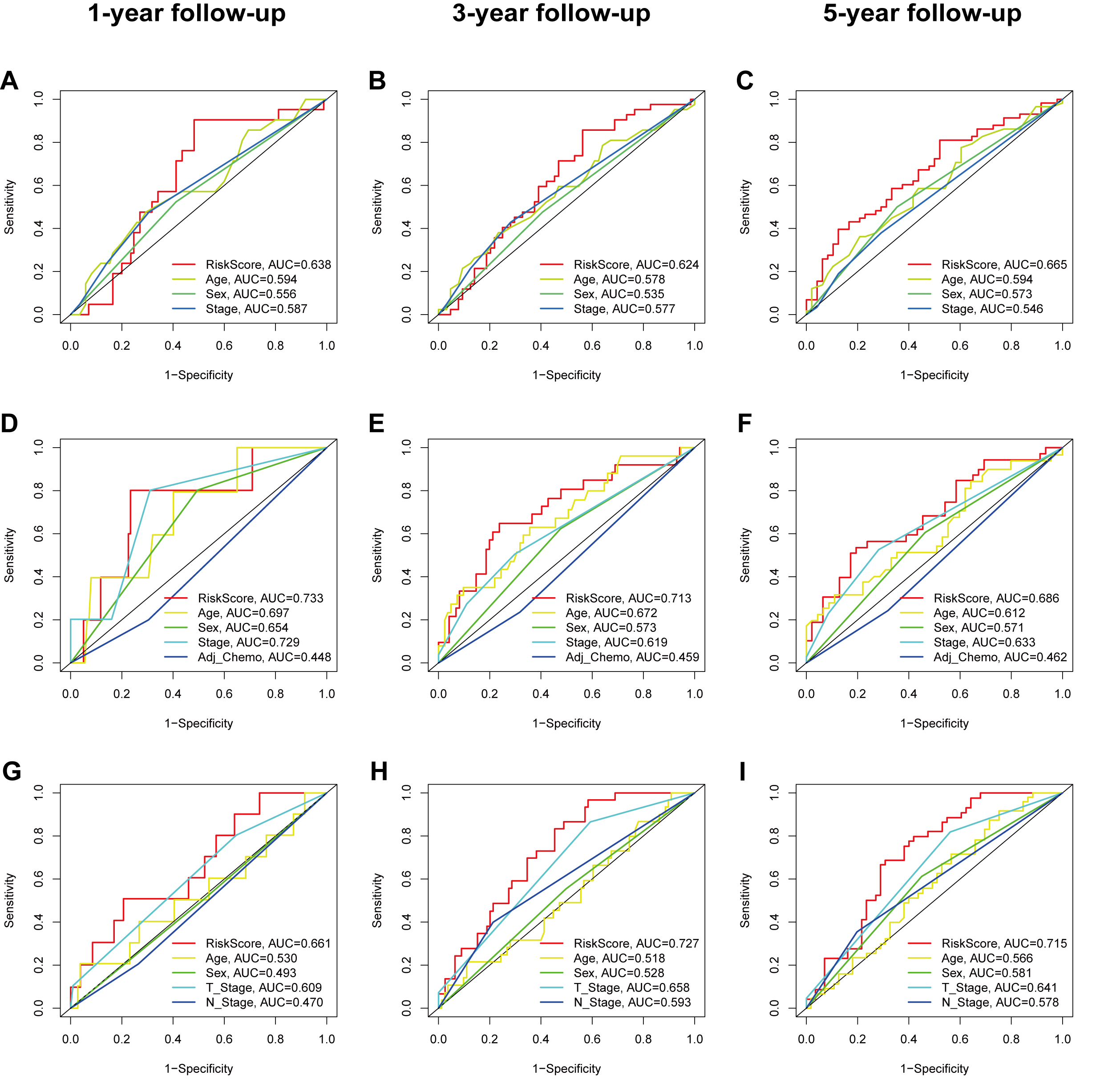

Supplement: Supplementary Figure 7 — The ROC curves of the risk score model and clinical factors at 1, 3, and 5-year follow-up. (A-C) ROC analysis of the COMAR risk score, age, sex, and tumor stage on the prognosis at 1-year (A), 3-year (B) and 5-year (C) follow-up in GSE37745 cohort. (D-F) ROC analysis of the COMAR risk score, age, sex, tumor stage and adjuvant chemotherapy on the prognosis at 1-year (D), 3-year (E) and 5-year (F) follow-up in GSE42127 cohort. (G-I) ROC analysis of the COMAR risk score, age, sex, T stage and N stage on the prognosis at 1-year (G), 3-year (H) and 5-year (I) follow-up in GSE50081 cohort. [file Image7.tif]

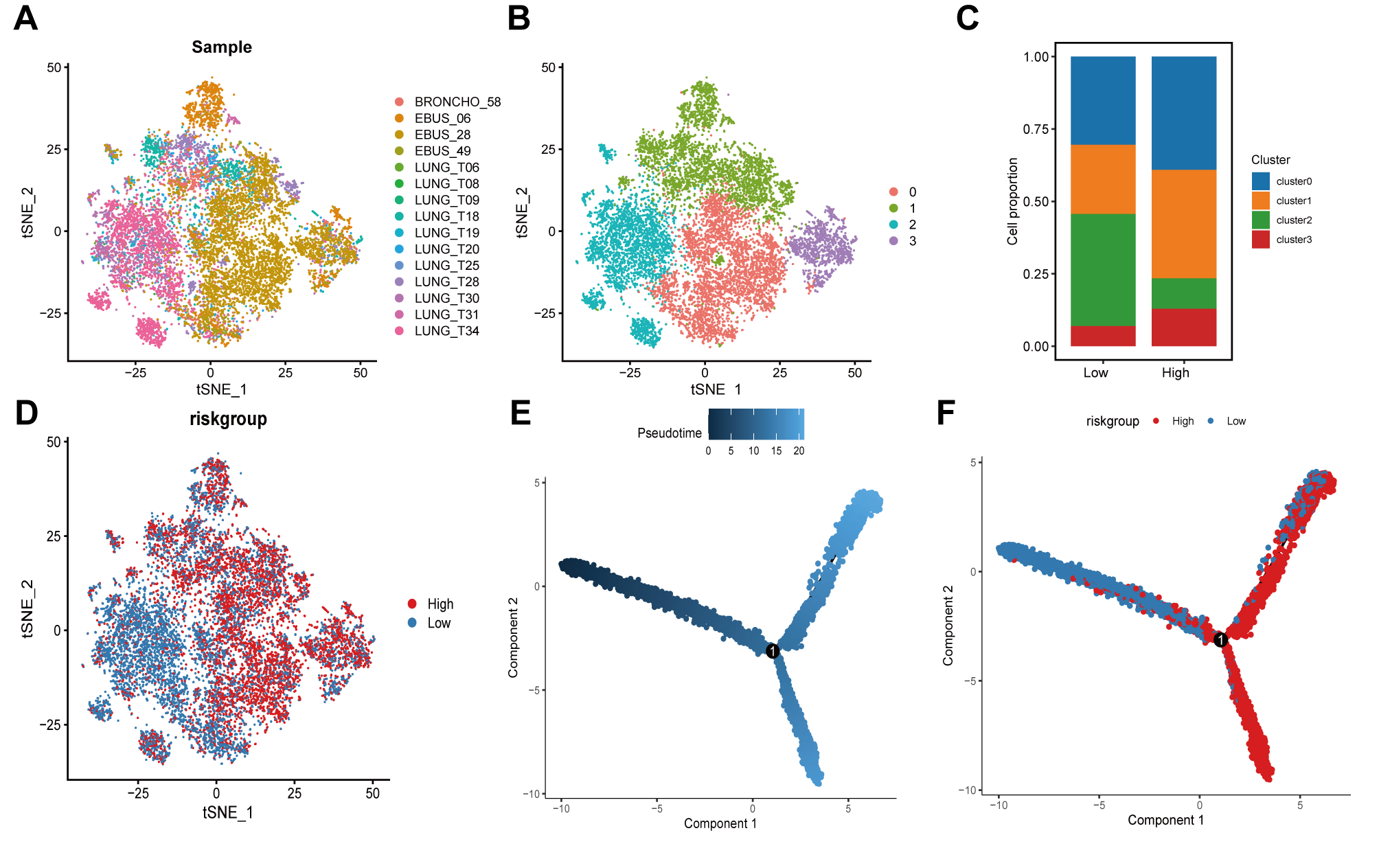

Supplement: Supplementary Figure 8 — Pseudotime analysis of the scRNA-seq data. (A) The TSNE plot of all the samples after removing the batch effect of malignant tumor cells. Different colors represent different samples. (B) The TSNE plot showing different subgroups of malignant tumor cells. Different colors represent different subgroups of tumor cells. (C) Percentage chart showing the percentages of different subgroups of tumor cells in the low-risk score and high-risk score groups. (D) The TSNE plot showing the calculation results of the risk score for malignant tumor cells. Each dot represents a tumor cell. The red dots represent cells in the high-risk group, whereas the blue dots represent cells in the low-risk group. (E) Temporal differences in the differentiation of malignant tumor cells. Different colors represent different pseudotimes. (F) Differentiation of cells in the high- and low-risk score groups. Different colors represent different cell groups. [file Image8.tif]

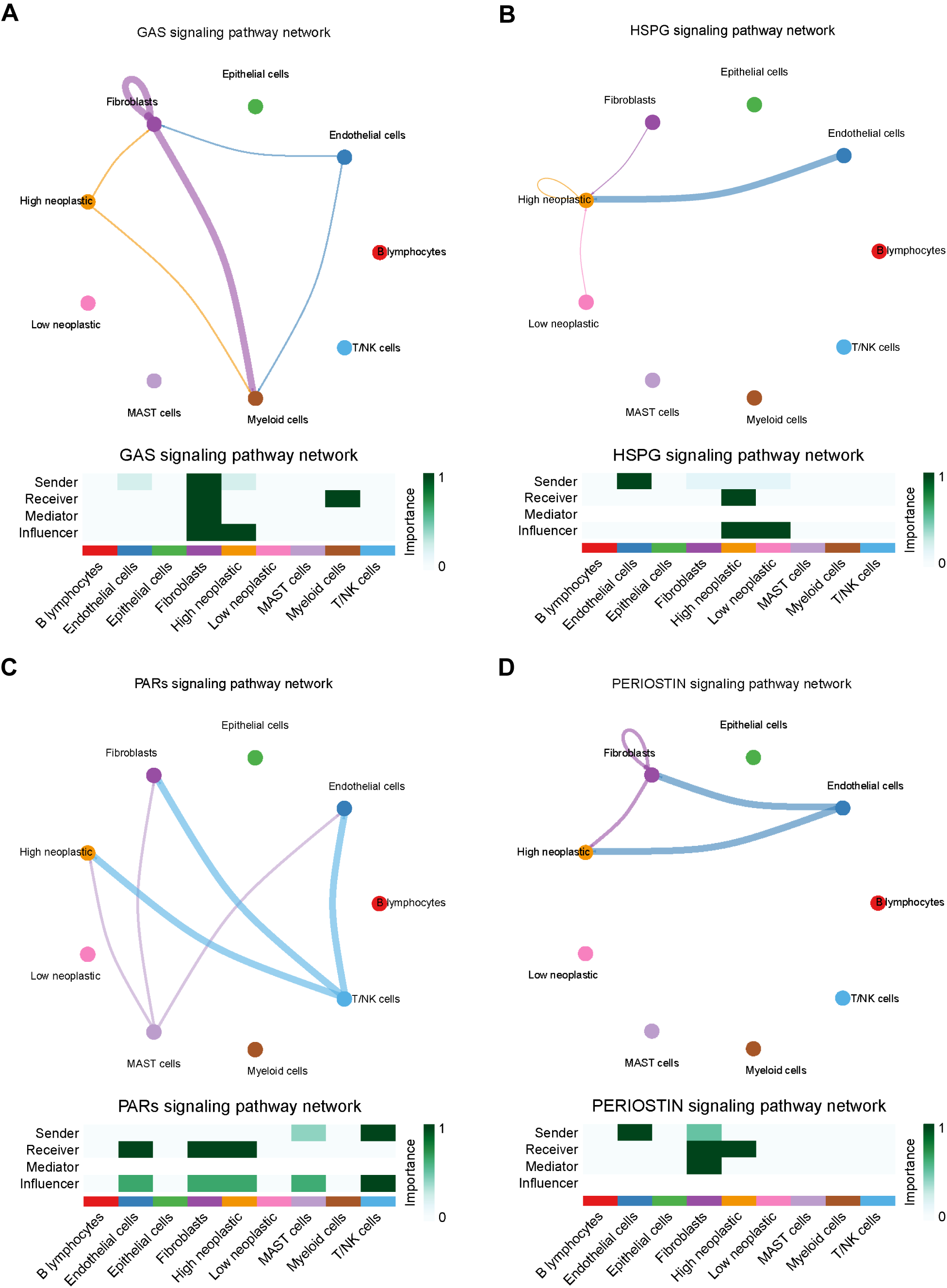

Supplement: Supplementary Figure 9 — Differences in cellular communication between the high- and low-risk score groups. (A) Tumor cells in the high-risk score group strongly communicate with myeloid cells and fibroblasts through the GAS pathway. (B) Tumor cells in the high-risk score group strongly communicate with endothelial cells through the HSPG pathway. (C) Tumor cells in the high-risk score group strongly communicate with T/NK cells through the PAR pathway. (D) Tumor cells in the high-risk score group strongly communicate with endothelial cells and fibroblasts through the PERIOSTIN pathway. [file Image9.tif]

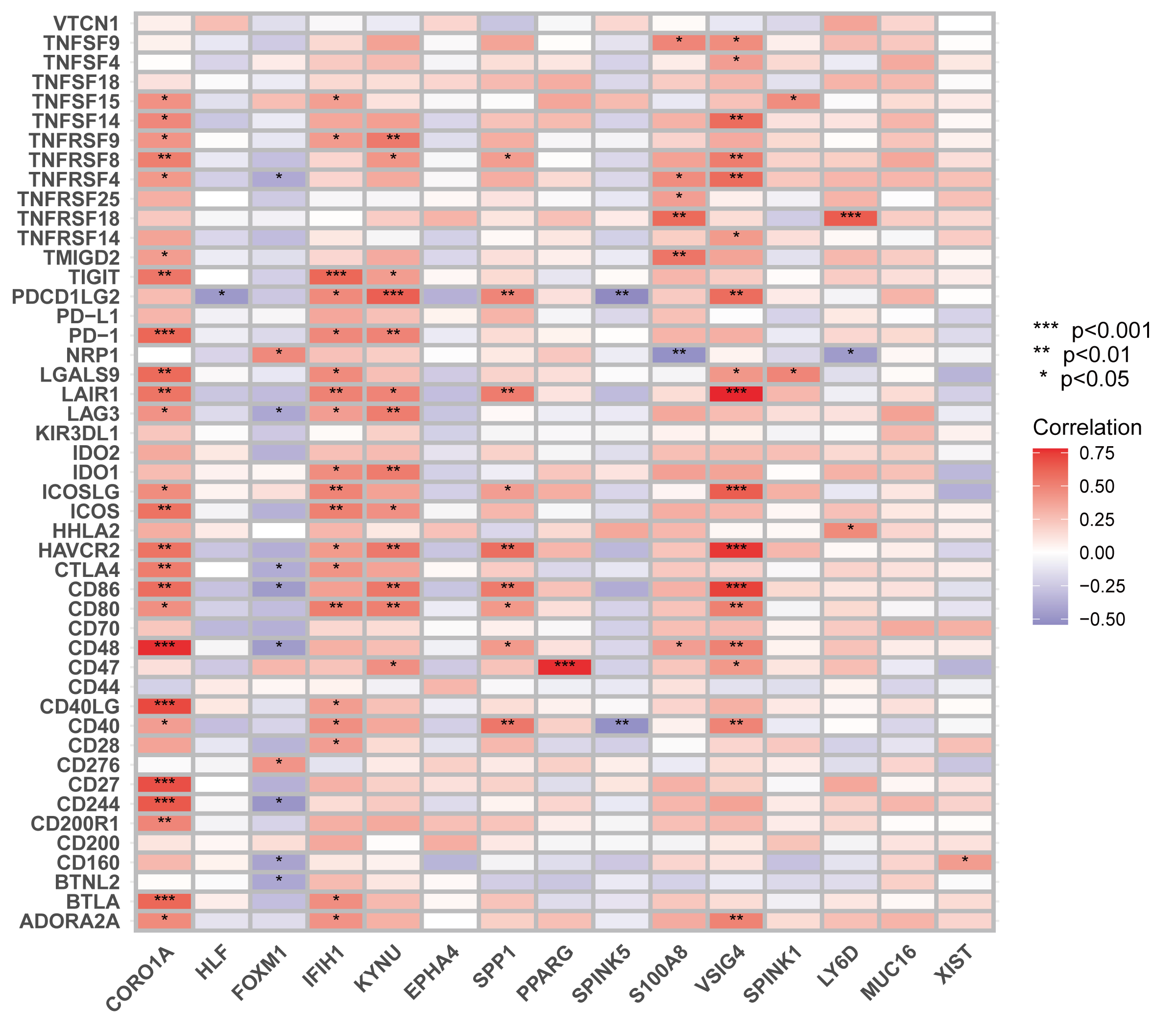

Supplement: Supplementary Figure 10 — The correlation analysis between the expression of the 15 genes and immune checkpoints in LUAD. [file Image10.tif]
